# Dual threshold optimization and network inference reveal convergent evidence from TF binding locations and TF perturbation responses

**DOI:** 10.1101/gr.259655.119

**Published:** 2020-03

**Authors:** Yiming Kang, Nikhil R. Patel, Christian Shively, Pamela Samantha Recio, Xuhua Chen, Bernd J. Wranik, Griffin Kim, R. Scott McIsaac, Robi Mitra, Michael R. Brent

**Affiliations:** 1 Center for Genome Sciences and Systems Biology, Washington University School of Medicine, St. Louis, Missouri 63110, USA;; 2 Department of Computer Science and Engineering, Washington University, St. Louis, Missouri 63130, USA;; 3 Department of Genetics, Washington University School of Medicine, St. Louis, Missouri 63110, USA;; 4 Calico Life Sciences LLC, South San Francisco, California 94080, USA

## Abstract

A high-confidence map of the direct, functional targets of each transcription factor (TF) requires convergent evidence from independent sources. Two significant sources of evidence are TF binding locations and the transcriptional responses to direct TF perturbations. Systematic data sets of both types exist for yeast and human, but they rarely converge on a common set of direct, functional targets for a TF. Even the few genes that are both bound and responsive may not be direct functional targets. Our analysis shows that when there are many nonfunctional binding sites and many indirect targets, nonfunctional sites are expected to occur in the *cis*-regulatory DNA of indirect targets by chance. To address this problem, we introduce dual threshold optimization (DTO), a new method for setting significance thresholds on binding and perturbation-response data, and show that it improves convergence. It also enables comparison of binding data to perturbation-response data that have been processed by network inference algorithms, which further improves convergence. The combination of dual threshold optimization and network inference greatly expands the high-confidence TF network map in both yeast and human. Next, we analyze a comprehensive new data set measuring the transcriptional response shortly after inducing overexpression of a yeast TF. We also present a new yeast binding location data set obtained by transposon calling cards and compare it to recent ChIP-exo data. These new data sets improve convergence and expand the high-confidence network synergistically.

Mapping the circuitry by which cells regulate gene expression is a fundamental goal of systems biology. Such maps would facilitate a broad spectrum of research programs, much as maps of intermediary metabolism and genome sequences have. Transcriptional regulation has multiple layers and component types, including sensors and signal transduction cascades. The bottom layer of transcriptional regulation, which acts directly at the genome, features sequence-specific DNA-binding proteins known as transcription factors (TFs). Signaling cascades often change the activity levels of specific TFs—the extent to which they exert their regulatory potential on their target genes—via mechanisms that affect TFs’ abundance, localization, noncovalent interactions, or covalent modifications. To map and model transcriptional regulation as a whole, we must know which genes each TF regulates or has the potential to regulate when activated.

A map of an organism's TF network would have powerful applications. It could be used to infer the effects of specific signals, drugs, or environments on the activity levels of TFs by analyzing their effects on gene expression (Liao et al. 2003; Tran et al. 2005; Boorsma et al. 2008; Balwierz et al. 2014). It could be used to predict the significance of naturally occurring genome variants in TFs or TF binding sites (TFBSs). It could also be used to design genome edits in TFs or TFBSs to achieve a desired transcriptional state or behavior (Cahan et al. 2014; Michael et al. 2016; Rackham et al. 2016). Crucial to all of these applications is the distinction between the direct functional targets of a TF—the genes it regulates because it binds to their *cis*-regulatory DNA—and its indirect targets, which are regulated via intermediary proteins. For example, a mutation inactivating a binding site for a TF in the *cis*-regulatory DNA of one of its direct targets will affect the relationship between the TF and its direct target. However, a mutation in a nonfunctional binding site which happens to lie in the *cis*-regulatory DNA of an indirect target will not affect the relationship between the TF and its indirect target.

In this paper, we analyze previously published and newly described genome-wide data sets (Table 1) with both standard and novel analytic techniques to reveal the current state-of-the-art in identifying the direct, functional targets of a TF. The data sets we focus on are those that aim to determine the binding locations of TFs and those that attempt to measure the transcriptional response to perturbations of TF activity, such as overexpressing the TF or deleting the gene that encodes it. The binding location data are derived from either chromatin immunoprecipitation (ChIP) or transposon calling cards (Wang et al. 2008; Ryan et al. 2012; Mayhew and Mitra 2016; Shively et al. 2019).

**Table 1.**
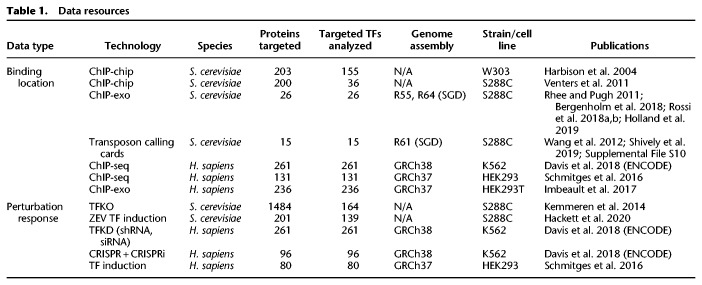
Data resources

Yeast data sets on TF binding locations and TF perturbation-responses are more complete than those of any other eukaryote, and yeast has a simpler genome with more localized regulatory DNA. For those reasons, we start by focusing on yeast. In addition to evaluating data sets and experimental and analytic methods, we construct a preliminary map of the yeast TF network by integrating the best available binding and perturbation response data sets. For model invertebrates, there are large data sets on TF binding location (Brown and Celniker 2015; Kudron et al. 2018), but there are currently no comparable data sets on the responses to TF perturbations. Such data are available, however, for human cell lines. We analyze large data sets on human K562 cells (The ENCODE Project Consortium 2012; Sloan et al. 2016) and HEK293 cells (Schmitges et al. 2016), producing TF networks for each cell type.

## Results

### Simple comparison of yeast ChIP-chip to expression profiles of TF deletion strains yields few high-confidence regulatory relationships

#### Comprehensive binding and perturbation response data sets are available for yeast TFs

In 2004, Harbison et al. assayed the binding locations of all yeast TFs by using ChIP-chip (Harbison et al. 2004). In 2007, Hu et al. published gene expression data on yeast strains in which each nonessential yeast TF was deleted (Hu et al. 2007). This made it possible to estimate the fraction of binding events that are functional, and Hu et al. remarked on how small that fraction is—about 3%–5% in their data. In 2014, Kemmeren et al. published a second such data set, which benefited from newer technology and the hindsight afforded by the earlier study (Kemmeren et al. 2014). In this section, we focus on the Kemmeren TF knockout (TFKO) data because it demonstrates better agreement with the Harbison ChIP data, on average.

#### Most bound genes in the Harbison ChIP data are not responsive in the TFKO data

We began by calculating the response rate of bound genes for each TF—the fraction of bound genes that are differentially expressed in the TFKO strain, relative to the wild type (WT). The microarrays used by Harbison et al. in their ChIP-chip study contained one probe for each promoter, so their analysis yielded a simple *P*-value for whether each promoter is bound. We eliminated from further consideration the 16 TFs that were not called as bound to any promoter. For the TFKO data, we used the authors’ statistical analysis and considered a gene differentially expressed if its *P*-value (adjusted for multiple comparisons) was <0.05. We eliminated from further consideration any TF whose knockout resulted in no significant changes, as well as the 32 TFs whose reported expression level in the strain lacking the TF was more than one half its reported level in the WT. This can happen when the wild-type expression level of the TF is near or below the detection limit of the microarray.

**Figure 1.**
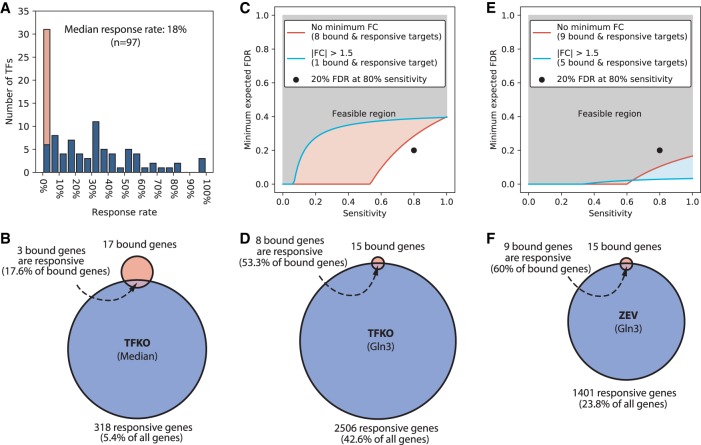
Overlap between bound and responsive gene sets. (*A*) Distribution of the response rates of TFs (fraction of bound genes that respond to TF perturbation) in the Harbison binding and Kemmeren TFKO data sets. Stacked orange bar indicates the number of TFs with response rates of exactly 0. Binding threshold is *P* < 0.001 and response threshold is *P* < 0.05, as recommended in the original publications, with no minimum fold change. (*B*) Median numbers of bound genes (17), perturbation-responsive genes (318), and intersection size (3), when comparing the ChIP-chip data to the TFKO perturbation-response data. Thresholds are as in *A*. (*C*) Minimum expected FDR as a function of sensitivity for TF Gln3, when comparing ChIP to TFKO. Genes are counted as responsive if they have adjusted *P* < 0.05 (blue line) or adjusted *P* < 0.05 and fold change >1.5 (salmon line). Eighty percent sensitivity with 20% FDR is not attainable at either threshold, when comparing ChIP to TFKO. (*D*) The bound set, responsive set, and intersection for Gln3, when comparing ChIP to TFKO. (*E*) Minimum expected FDR, as a function of sensitivity, with moderate and tight thresholds for responsiveness, when comparing ChIP to ZEV15. Eighty percent sensitivity with 20% FDR is attainable at either threshold. (*F*) The bound set, responsive set, and intersection for Gln3, when comparing ChIP to ZEV15.

Figure 1A shows a histogram of the results. The median response rate for bound genes was 18%. The mode was 0%; 25 of the 97 TFs (26%) had both bound targets and responsive targets, but none of the bound targets were responsive. Only 17 TFs (18%) had a response rate above 50%. Tightening the statistical significance threshold for responsiveness lowers the response rate further, while tightening the threshold for binding causes very few genes to be classified as bound and responsive (Supplemental Fig. S1A–C). Thus, these data do not support the notion that most binding is functional. The low response rate of bound genes cannot be explained by saying that the TFs are inactive in the conditions tested, since the median number of genes that respond with *P* < 0.05 is 318. A lot of genes respond, but they are not the bound genes.

#### Many genes that are both bound and responsive in previously published data are probably not direct functional targets

Given that available data suggest most binding sites are nonfunctional, a logical procedure for finding the direct functional (DF) targets is to take the intersection of the genes bound by each TF with the genes that respond to perturbation of that TF, a procedure we refer to as the *intersection algorithm*. It is important to keep in mind, however, that most responsive genes are not bound. Comparing the ChIP data with the TFKO data, the median fraction of responsive genes that are bound is 1% (Fig. 1B). Thus, most of the responsive genes are indirect targets. Furthermore, it is reasonable to assume that the distribution of indirect targets among all genes is independent of the distribution of nonfunctional binding sites, or at least that nonfunctional binding sites do not systematically avoid the promoters of indirect targets. This suggests that some of the indirect targets also have nonfunctional binding sites. These genes would be false positives of the intersection algorithm—genes that are bound and responsive but are not responsive *because* they are bound.

In Supplemental Box S1, we derive a new lower bound on the expected false discovery rate (FDR) of the intersection algorithm, as a function of its sensitivity (the fraction of direct functional targets that are in the intersection) and four other variables: the number of bound genes, |*B*|, the number of responsive genes, |*R*|, the number of bound and responsive genes, |B∩R|, and the total number of genes assayed, |*G*|.

$$E[FDR]\geq \frac{\max(0,\;|B|-|R\cap B|/\mathrm{Sn})\max(0,\;|R|-|R\cap B|/\mathrm{Sn})}{\left|G||B\cap R\right|}$$

The formula shows that, if a large fraction of bound genes is not responsive and a large fraction of responsive genes is not bound, the intersection procedure cannot have both high sensitivity and low false discovery rate. For example, Fig. 1C shows the relationship between sensitivity and expected FDR for a fairly typical TF, Gln3, based on the Harbison ChIP data and the TFKO response data. The blue and red lines form the boundaries between the feasible and infeasible regions for two different response thresholds. They are calculated by varying the sensitivity and using the formula shown above to calculate the corresponding lower bound on the expected FDR. A reasonable minimum accuracy criterion for a procedure aimed at finding the DF targets of a TF is that it have sensitivity ≥80% (it detects at least 80% of the DF targets) and an FDR ≤ 20%. However, that is not possible for Gln3, using these two data sets (Fig. 1C, black dot). This is because the fraction of Gln3-bound genes that are responsive to the Gln3 perturbation (53%) is only a little more than the fraction of all genes that are responsive to the Gln3 perturbation (43%; Fig. 1D). The 80–20 criterion is achievable for only 43 TFs. Supplemental Figure S2 shows the effect of varying the significance thresholds for binding and response.

The FDR lower bound does not guarantee any maximum FDR for the intersection algorithm. In fact, of the 43 TFs that could possibly achieve the 80–20 criterion in the ChIP-TFKO comparison, only 27 have an intersection that is significantly larger than would be expected by chance (hypergeometric *P* < 0.01, not adjusted for multiple testing). Conversely, three TFs that passed the *P* < 0.01 criterion failed the 80–20 criterion. If we define “TF with acceptable convergence” to be one that could pass the 80–20 criterion and has a larger overlap between bound and responsive targets than would be expected for randomly selected gene sets, then there are 27 acceptable TFs with 448 interactions regulating 366 target genes. If we take this to be our network map, ∼85% of TFs do not have acceptable convergence, so they have no high confidence targets, while 94% of genes have no identifiable regulator. In summary, using the simple intersection algorithm with just these two data sets does not produce anything like a complete TF network map.

### Comparing yeast ChIP-chip data to expression profiling shortly after TF induction enlarges the map

Recently, some of us released a data set in which the expression of nearly every yeast TF was induced from a very low level to a high level (http://idea.research.calicolabs.com) (Hackett et al. 2020). This was accomplished by expressing ZEV, an estradiol-activated artificial TF, and replacing the promoter of the gene to be induced with a ZEV-responsive promoter (McIsaac et al. 2013, 2014). (Some of the TFs were induced using an earlier iteration of the artificial TF called GEV [McIsaac et al. 2011], but we refer to the data set as ZEV for convenience.) Gene expression profiles were measured before induction and at 5, 10, 15, 20, 30, 45, and 90 min after inducing the expression of a natural yeast TF with estradiol. We reasoned that genes that respond rapidly might be enriched for direct targets of the induced TF, since there would be limited time for intermediary proteins to be transcribed and translated. If the responders were enriched for direct targets, the number of TFs showing acceptable convergence might increase, expanding the network map. In general, the expression profiles taken 15 min after TF induction (ZEV15) were most enriched for bound genes, so we focus on the 15-min time point for the remainder of the paper (Supplemental Fig. S3). For a detailed description of the strains, experiments, and analysis, see Hackett et al. (2020).

**Figure 2.**
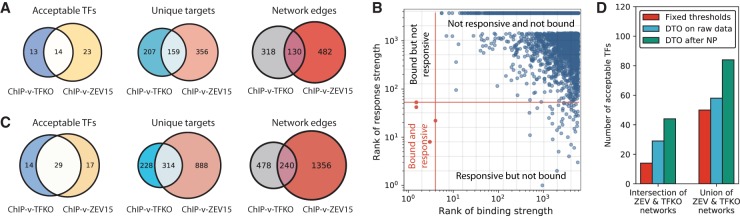
Dual threshold optimization (DTO) and network inference in yeast. (*A*) Numbers of acceptable TFs, unique target genes, and network edges, when comparing Harbison ChIP data to TFKO or ZEV15 response data. “Unique targets” are genes that are in the bound-responsive intersection of at least one acceptable TF and thus are plausible direct functional targets. Edges connect acceptable TFs to the genes in their bound-responsive intersection. The ZEV15 response data yield more acceptable TFs, more unique targets, and more regulatory edges. (*B*) Illustration of DTO algorithm. Each dot represents one gene. Red lines indicate the chosen (optimal) thresholds for binding (vertical red line) and regulation (horizontal red line). The *lower left* quadrant, relative to the red lines, contains the bound and responsive genes, which are presumed to be direct functional targets (red dots). Gray lines indicate some of the other possible thresholds on binding or response, and locations where the gray lines cross are possible combinations of binding and response thresholds, each of which is evaluated by the DTO algorithm. (*C*) Numbers of acceptable TFs and unique target genes for comparison of Harbison ChIP binding data to TFKO or ZEV15 response data, after dual threshold optimization. The requirement that the overlap between the bound and responsive targets be greater than chance at *P* < 0.01 was checked by comparing the nominal hypergeometric *P*-value for the overlap to a null distribution obtained by running dual threshold optimization on 1000 randomly permuted binding and response data sets. DTO increases the network size, relative to using fixed significance thresholds. ZEV15 still yields more acceptable TFs, regulated genes, and regulatory interactions than TFKO. (*D*) Comparison of TFKO and ZEV15 networks derived from fixed thresholds, DTO on raw gene expression, and DTO on gene expression data processed by NetProphet 2.0. The use of DTO on the raw expression data (blue bars) increases the size of both the intersection of the ZEV15 and TFKO (*left* bar grouping) and their union (*right* bar grouping). Postprocessing with NetProphet 2.0 (green bars) further increases the number of acceptable TFs.

The TF Gln3, which could not achieve 80% sensitivity with 20% expected FDR in the ChIP-TFKO comparison (Fig. 1C), can in the ChIP-ZEV15 comparison (Fig. 1E). The reason is that the number of responsive genes has decreased from 43% of all genes to 24%, at the same time that the response rate of bound genes *increased* from 53% to 60% (Fig. 1D,F). Across all TFs, the ChIP-ZEV15 comparison identified 37 acceptable TFs, 23 of which had not been identified in the ChIP-TFKO comparison (Fig. 2A). The ChIP-ZEV15 comparison significantly expanded the network map. Still, >72% of TFs do not show acceptable convergence in either data set and hence have no identifiable targets, while >87% of genes have no identifiable regulators.

### Dual threshold optimization expands the TF network map

A possible limitation of the previous analyses is their sensitivity to the statistical significance thresholds used to determine which genes are bound and which are responsive. The statistics are calculated separately for the binding and response data sets, and statistical significance thresholds are, by their nature, arbitrary. Furthermore, statistically significant levels of binding or perturbation response might not be biologically significant. For example, a TF may bind a site consistently in the ChIP data even though the fractional occupancy of the site is too low to detectably affect transcription.

To address these problems, we developed dual threshold optimization (DTO), a method that sets the binding and response thresholds by considering both data sets together. DTO chooses, for each TF, the pair of (binding, response) thresholds that minimizes the probability that the overlap between the bound and responsive sets results from random gene selection (Fig. 2B). For this analysis, we ranked all genes by their absolute log fold change in the ZEV15 data and, separately, by their negative log *P*-value in the ChIP-chip data. We then chose the pair of (binding, response) rank thresholds that minimized the nominal hypergeometric *P*-value of the overlap between bound and responsive gene sets. The only constraint on the thresholds chosen was that the *P*-value for the ChIP data could not exceed 0.1. To test the significance of the overlap at the chosen thresholds, we randomly permuted the assignment of binding and response signals to genes 1000 times and ran DTO on each random permutation (see Supplemental Methods for details).

After DTO, we applied the same acceptable convergence criteria as before—the bound-responsive overlap must be significant (*P* < 0.01, permutation-based) and 20% FDR at 80% sensitivity must be theoretically achievable. DTO expanded the network map again (Fig. 2C). Combining the results from TFKO and ZEV15, 60 TFs showed acceptable convergence. For these 60, the bound-responsive overlap contained 2074 regulatory interactions involving 1430 unique target genes. The number of TFs that are acceptable in both response data sets, 29, now exceeds the number that are acceptable in either of the data sets alone (TFKO: 14, ZEV15: 17). In this map, ∼33% of TFs have at least one target and ∼24% of genes have at least one regulator. The maps based on DTO of TFKO and ZEV15 data are provided as Supplemental Files S1 and S2.

### Processing yeast gene expression data with a network inference algorithm further expands the map

There are many algorithms that attempt to infer TF-target relationships by processing gene expression data but not binding location data (e.g., Margolin et al. 2006; Faith et al. 2007; Huynh-Thu et al. 2010; Haury et al. 2012; Greenfield et al. 2013; Haynes et al. 2013; Roy et al. 2013; Kang et al. 2018). Typically, they assign a confidence score to each possible TF-target interaction. If all possible targets of a TF are ranked according to their score, DTO can be applied to compare this ranking to binding location data. As long as the network inference algorithm does not use any binding data, DTO can provide independent, convergent evidence. There are also network inference algorithms that weigh and integrate data sources, including gene expression and TF binding location data or curated sources influenced by binding data (e.g., Siahpirani and Roy 2017; Wang et al. 2018; Castro et al. 2019). These algorithms are not suitable for our current purpose, which is to assess the convergence of *independent* evidence from gene expression and binding location data.

To test this idea, we focused on our lab's network inference algorithm, NetProphet 2.0 (Kang et al. 2018). A major component of the NetProphet score is the degree to which the target gene responds to direct perturbation of the TF. However, it also considers the degree to which the mRNA level of the TF is predictive of the mRNA level of the potential target, across many different perturbations. NetProphet also makes use of two other ideas: (1) that coregulated genes tend to have similar sequence motifs in their promoters; and (2) that DNA-binding domains with similar amino acid sequences tend to bind similar motifs. It does not use any data on TF binding location, either directly or indirectly.

We built separate NetProphet networks using the TFKO and ZEV data (Methods). For TFKO, we input three wild-type expression profiles and the complete set of 1484 expression profiles from strains lacking one gene; some of the deleted genes encode TFs, but others encode other putative regulatory proteins, such as kinases and phosphatases. For ZEV, we used 590 expression profiles from 15 , 45 , or 90 min postinduction. We then ranked the potential targets of each TF by their NetProphet scores and ran dual threshold optimization, treating the NetProphet score as we did the perturbation response strength. Combining the results from NetProphet applied to TFKO and ZEV data, dual threshold optimization yielded 84 TFs (46%) with acceptable convergence (Fig. 2D). For these TFs, the bound-responsive intersection had 2153 regulatory interactions involving 1327 unique target genes (23%) (Supplemental Fig. S4A,B). The number of TFs that are acceptable in both perturbation data sets, 44, is now much larger than the number that are acceptable in either data set alone (TFKO: 22, ZEV: 18). Supplemental Files S3 (TFKO) and S4 (ZEV) contain the regulatory edges for each acceptable TF. Results from comparing binding data to output from three other network inference algorithms, Inferelator (Greenfield et al. 2013), GENIE3 (Huynh-Thu et al. 2010), and MERLIN (Roy et al. 2013), can be found in Supplemental Figure S4C.

Running NetProphet on gene expression data and feeding the result into dual threshold optimization has enlarged the map, but it is still smaller than what is generally expected for the complete yeast TF network. To improve it further, we need binding data that are more accurate or more specifically focused on functional binding.

### Without network inference, data on human cell lines yield a few acceptable TFs

The ENCODE Project (The ENCODE Project Consortium 2012) has produced a wealth of data on human cell lines, including 743 TF ChIP-seq experiments and 391 RNA-seq experiments following knockdown of a TF by siRNA or shRNA (TFKD), or by CRISPR interference (Gilbert et al. 2014) or CRISPR knockout (CRISPRi + CRISPR KO). In K562 cells, 42 TFs have both ChIP-seq and TFKD data, while 45 TFs have both ChIP-seq and CRISPRi or CRISPR KO data. We focus on this K562 data, as it is by far the biggest relevant data set.

We considered two ways of assigning ChIP-seq peaks to the genes they potentially regulate. The first is the traditional approach of choosing a fixed interval around the transcription start site (TSS)—we used 10 kb upstream to 2 kb downstream. The second is to take a small proximal promoter region (TSS −500 bp to +500 bp) along with enhancer regions that have been identified and assigned to the target gene in the GeneHancer database (Fishilevich et al. 2017). GeneHancer uses a variety of data types, including predicted and ChIP-based TF binding sites, enhancer RNAs, histone marks, chromosome conformation, and *cis*-eQTLs. We used only the ‘elite’ enhancers and ‘elite’ associations, each of which is supported by at least two sources of evidence. Ninety-one percent of the ‘elite’ enhancers were supported by evidence from ENCODE, much of which comes from K562 cells. The enhancer-based approach generally yielded one or two more TFs with acceptable convergence than the fixed interval approach, so we used the enhancers in subsequent analyses.

**Figure 3.**
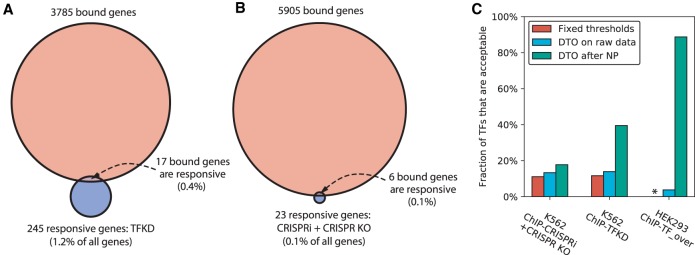
Network inference with dual threshold optimization in human cell lines. (*A*) Medians of number of bound genes, number of perturbation-responsive genes, and number of genes that are both bound and responsive, when comparing ENCODE K562 ChIP-seq data to ENCODE TFKD data. Excludes TFs with either no bound genes or no responsive genes. Binding threshold is *P* < 0.05 and response threshold is *P* < 0.05 with no minimum fold change. (*B*) Comparison of ENCODE K562 ChIP-seq data and ENCODE CRISPRi + CRISPR KO data, as in *A*. (*C*) Comparison of human networks derived from fixed thresholds, dual threshold optimization on raw perturbation-response data, and DTO on perturbation-response data processed by NetProphet 2.0. The vertical axis is the number of TFs showing acceptable convergence divided by the number that were both ChIPped and perturbed (K562: ChIP-CRISPRi + CRISPR KO = 45, K562: ChIP-TFKD = 43, HEK293: ChIP-TF_over = 80). Asterisk indicates that no fixed threshold analysis for HEK293 is available due to the lack of response *P*-values.

Unlike the yeast array data, the human sequencing data yielded many more bound than responsive genes (Fig. 3A,B). Among the TFs that had at least one bound and one responsive gene, seven (TFKD) and seven (CRISPRi + CRISPR KO) had no genes that were both bound and responsive. The median response rate for bound genes was <0.5%. In a fixed-threshold intersection with K562 ChIP-seq data, TFKD and CRISPRi + CRISPR KO each yielded five TFs with acceptable convergence. We then ran dual threshold optimization limiting the bound and responsive gene sets to have *P* ≤ 0.1; such limits are necessary because DTO occasionally chooses implausible thresholds, such as counting all genes as responsive. Among all TFs with both binding and response data, TFKD yielded 14% acceptable TFs (6/43) and CRISPRi + CRISPR KO yielded 13% (6/45), a slight improvement over fixed-threshold intersections (Fig. 3C, left and center).

We also analyzed a data set on 88 human GFP-tagged C2H2 zinc finger TFs with matched ChIP-seq data and response-to-overexpression data in HEK293 cells (Schmitges et al. 2016). Using DTO on the ChIP-seq and differential expression data and limiting the total number of responsive genes to 300,000, three of 88 TFs showed acceptable convergence (Fig. 3C, right).

### Processing human data through network inference algorithms greatly increases the number of acceptable TFs

We ran NetProphet 2.0 on both the K562 data (TFKD and CRISPRi + CRISPR KO) and the HEK293 data followed by DTO, limiting the total set of responsive genes to those with the top 500,000 (K562) or 300,000 (HEK293) NetProphet scores (see Supplemental Methods for details). Among the TFs that were both perturbed and ChIPped, the number showing acceptable convergence increased from six to eight (K562 CRISPRi + CRISPR KO), from six to 17 (K562 TFKD), and from three to 71 (HEK293 overexpression) (Fig 3C). Comparable results for other network inference algorithms are shown in Supplemental Figure S4D. NetProphet and other inference algorithms can also infer targets for TFs that have not been directly perturbed, by exploiting correlation between the expression of the TF and its targets when other TFs are perturbed. Processing all the perturbation response data and evaluating only on the nonperturbed TFs, we found that the Inferelator scores yielded the largest number of TFs with acceptable convergence (Supplemental Fig. S4E). This is not surprising, since NetProphet weighs the response to direct perturbation heavily in its score. This suggests that, for TFs that have not been directly perturbed, Inferelator is the best choice of analysis tool.

We also compared the output of NetProphet 2.0 when run on HEK293 perturbation response data to a recently published ChIP-exo data set (Imbeault et al. 2017) focusing on KRAB zinc finger TFs. ChIP-exo (Rhee and Pugh 2011, 2012; Perreault and Venters 2016; Rossi et al. 2018b) is a variant of ChIP-seq in which the affinity-purified chromatin is digested by an exonuclease, leaving much smaller pieces that are partially protected by protein. Of the 27 TFs that were in both perturbation and ChIP-exo data sets, 20 showed acceptable overlap with NetProphet scores. For the same 27 TFs, using the previously described ChIP-seq yielded 24 TFs with acceptable convergence. This small difference may be due, in part, to the fact that the ChIP-exo experiments were done on a derivative cell line known as HEK293T.

### In yeast, newer ChIP data do not necessarily yield better convergence with perturbation response

**Figure 4.**
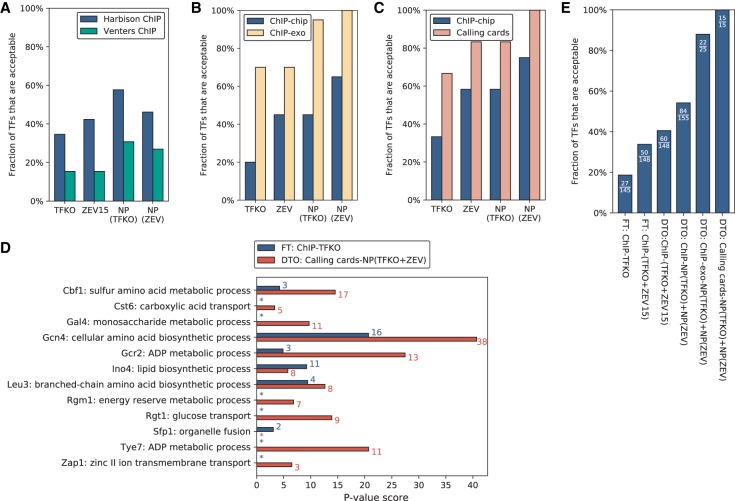
Generating a high-confidence yeast TF network. (*A*) Percentage of TFs showing acceptable convergence, when comparing the Harbison ChIP and Venters ChIP data on the same 26 TFs. Regardless of the perturbation data set or the processing by NetProphet 2.0, the Harbison ChIP data always yield more acceptable TFs. (*B*) Among the 20 TFs for which we have data in Harbison ChIP-chip, ChIP-exo, TFKO, and ZEV, the percentage that show acceptable convergence. Regardless of the perturbation data set or processing by NetProphet 2.0, ChIP-exo always yields more acceptable TFs. For both TFKO and ZEV, NetProphet postprocessing yields more acceptable TFs than raw differential expression. When NetProphet-processed ZEV data are compared to ChIP-exo data, all TFs show acceptable convergence. (*C*) Among the 12 TFs for which we have data in Harbison ChIP, calling cards, TFKO, and ZEV, the percentage that show acceptable convergence. When NetProphet-processed ZEV data are compared to calling cards, all TFs show acceptable convergence. (*D*) For each of the 12 TFs for which we have data in Harbison ChIP, calling cards, TFKO, and ZEV, the Gene Ontology (GO) term that is most strongly enriched in the TF's targets. Targets are determined either by simple intersection of the bound and responsive genes in Harbison ChIP and TFKO data, using fixed thresholds (blue) or by dual threshold optimization on calling cards data and output from NetProphet 2.0 run on the TFKO and ZEV expression data (red). The colored numbers indicate the number of target genes annotated to the most significant GO term. Asterisk indicates no GO enrichment with *P* < 0.01. (*E*) Among all TFs for which the indicated analyses can be carried out, the percentage that are acceptable in either TFKO or ZEV data or both. The fraction shows the number of acceptable TFs over the total number of TFs that could be analyzed. (FT) Fixed threshold, (DTO) dual threshold optimization.

To assess whether the age of the Harbison ChIP-chip data was responsible for some of its limitations, we analyzed a 2011 ChIP data set from Venters et al. (2011), which included 26 factors that were also ChIPped by Harbison and perturbed by TFKO and ZEV. The results did not improve on those of Harbison et al. (Fig. 4A).

### In yeast, ChIP-exo yields better convergence than traditional ChIP

We also ran DTO on ChIP-exo data from yeast (Rhee and Pugh 2011; Bergenholm et al. 2018; Rossi et al. 2018a,b; Holland et al. 2019). Twenty TFs had data in ChIP-exo, Harbison ChIP-chip, TFKO, and ZEV15, enabling all-way comparisons. Regardless of the perturbation-response data set, ChIP-exo showed acceptable convergence for more TFs than ChIP-chip did (Fig. 4B). (For the sixteen TFs with ChIP-exo data in four different growth conditions, we used the glucose-limited chemostat data as it gave the best results [dotted blue lines, Supplemental Fig. S5A,B].) After processing the ZEV perturbation-response data through NetProphet 2.0, all 20 TFs showed acceptable convergence (Fig. 4B).

### Transposon calling cards yields more acceptable TFs than traditional ChIP

Transposon calling cards is a method of determining TF binding locations by tethering a transposase to a TF, recovering the inserted transposons with their flanking sequences, and counting the insertions in a given genomic region. It does not require crosslinking, sonication, or affinity purification (Wang et al. 2011; Ryan et al. 2012; Mayhew and Mitra 2016). Here, we analyze previously published calling cards data on seven TFs (Wang et al. 2011; Shively et al. 2019) and new, never-before-analyzed data on eight TFs. Binding data from ChIP-chip and calling cards were compared to perturbation-response data from TFKO and ZEV15, using the 12 TFs present in all four data sets (Fig. 4C). In all comparisons, calling cards yielded substantially more acceptable TFs than ChIP-chip. This is particularly impressive given that the calling cards experiments were carried out in different growth conditions from the ZEV experiments—synthetic complete medium with galactose on agarose plates at room temperature versus minimal medium in phosphate-limited continuous-flow chemostats with glucose at 30°C (Hackett et al. 2020). Figure 4C also shows that, holding all other factors constant, ZEV was always better than TFKO and postprocessing by NetProphet was always beneficial. Lists of acceptable TFs and their bound and responsive targets for all calling cards analyses in Fig. 4C are provided as Supplemental Files S5–S8.

Figure 4D shows the −log *P*-value of the most significant Gene Ontology (GO) term for the predicted targets of each TF for which we have calling cards data, excluding GO terms that describe >300 or fewer than three genes. To highlight the progress reported here, results are shown for the best combination of experimental and analytic methods (DTO on calling cards data and NetProphet output after processing TFKO and ZEV 15-, 45-, and 90-min samples) compared to the simple intersection of bound and responsive genes using TFKO and ChIP-chip. For 10 of 12 TFs, the best combination of methods had a more significant GO term *P*-value, and the differences were large. For two of 12 (Ino4 and Sfp1), simple intersection had the more significant *P*-value, but the differences were smaller. The median −log_10_ *P*-value for the best combination of methods was 11.2, while that of simple intersection was 1.5. The best combination of methods assigned the top GO term to 117 target genes, whereas simple intersection assigned the top term to only 41 genes. For most TFs, the most significant GO term had a clear relationship to the known function of the TF. In some cases, the term selected is an immediate parent of the most familiar term associated with the TF. For example, Gcr2 (Glycolysis Regulation 2) is known as a regulator of genes encoding glycolytic enzymes. Its most significant GO term is “ADP metabolic process,” annotating 13 predicted Gcr2 targets, but 12 of those targets are also annotated with “glycolytic process,” a subcategory of “ADP metabolic process.” This can be seen in Supplemental Figure S6, which shows the top five GO terms for each TF.

Another way to look at the contributions of various methods is to plot the fraction of available TFs that show acceptable convergence, combining TFKO and ZEV, using each combination of methods described here (Fig. 4E). Only 15 TFs are currently available for calling cards and either ZEV15 or TFKO (12 for both), but analyzing these with DTO and NetProphet results in a much larger fraction of TFs being acceptable. This includes TFs that are not thought to be active in the ZEV or TFKO growth conditions, such as Gal4, presumably because ZEV overexpression of Gal4 significantly exceeds the number of Gal80 molecules available to bind and inactivate it. The second best percentage of TFs showing acceptable convergence was obtained by comparing NetProphet scores to ChIP-exo data (Fig. 4E).

### The combination of ZEV and calling cards greatly increases response rates

**Figure 5.**
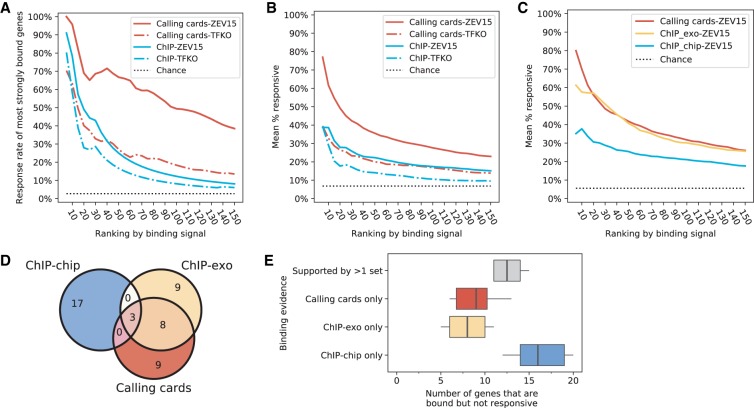
Comparison of yeast perturbation-response and binding data sets. (*A*) The fraction of most strongly Leu3-bound genes that are responsive to Leu3 perturbation, as a function of the number of most strongly bound genes considered. (*B*) Same as *A*, with response rates averaged across the 12 TFs for which Harbison ChIP, calling cards, TFKO, and ZEV data were available. (*C*) Same as *B*, with response rates averaged across the eight TFs for which Harbison ChIP, calling cards, ChIP-exo, and ZEV15 data were available. (*D*) Venn diagram for the 20 genes that are most strongly bound by Leu3 in each assay but not responsive to Leu3 perturbation (ZEV15). Only the top 20 nonresponsive genes ranked by their binding strengths are shown. (*E*) The analysis shown in *D*, applied to the eight TFs for which we have data in ChIP-chip, ChIP-exo, calling cards, and ZEV. The three colored box plots show the genes that are only bound in one of the three binding sets. The box plot in gray shows the genes with evidence in at least two binding sets.

We began this paper by observing that, using fixed threshold analysis of the TFKO and ChIP data, most binding appears to be nonfunctional. To revisit the question of functionality using ZEV15 and calling cards data, we plotted the fraction of bound genes that are responsive as a function of binding strength rank. Figure 5A shows that, for the TF Leu3, the combination of calling cards and ZEV15 gives much higher response rates than any of the other three combinations—ChIP-ZEV15, calling cards-TFKO, or ChIP-TFKO—regardless of binding strength. Nine out of the 10 most strongly bound and 48 out of 100 most strongly bound genes were responsive. To make the comparison between ZEV15 and TFKO fair, we fixed the number of Leu3-responsive genes in each perturbation data set to be the same. Thus, we labeled the 156 most strongly responsive genes in each data set as Leu3-responsive, because 156 was the minimum of the numbers of genes that were significantly differentially expressed in the two data sets for Leu3. Although the number of responsive genes in each data set was the same, a larger fraction of the ZEV15-responsive genes was bound, as compared to the TFKO-responsive genes. Figure 5B shows a similar plot of the average response rates at each binding threshold across the 12 TFs for which we have all four combinations of data sets. Again, the combination of ZEV15 and calling cards gives higher response rates at all binding thresholds. On average, the response rate of the 10 most strongly bound genes is 61.7%. Individual rank-response plots for the 11 other TFs present in all four data sets are shown in Supplemental Figure S7.

Figure 5C shows a direct comparison of binding strengths as assessed by calling cards, ChIP-exo, and ChIP-chip for the eight TFs for which we had data from all methods. Each binding data set was compared to ZEV15 data on the same TF. At the highest binding strengths, calling cards appears to be a bit more discriminating, but ChIP-exo catches up when 20 or more top binding targets are considered. Both calling cards and ChIP-exo greatly outperform ChIP-chip.

### Comparison of nonresponsive genes that are bound in each assay

Genes that appear to be bound by a TF but are not responsive to it could reflect false positives of the binding assay, nonfunctional binding sites, or genuinely bound genes that are not responsive because of network compensation, saturation, or other biological mechanisms (see Discussion). To estimate the contribution of false positives of the binding assays, we compared the bound but nonresponsive targets according to each assay, for the eight TFs for which we had all three assays (Fig. 5D). The nonresponsive genes that are bound in only one assay are more likely to be false positives than those that are supported by multiple assays. Binding at these genes could be supported by another assay at a level below the threshold we used for this analysis, so we cannot conclude that they are definitely false positives. We found that ChIP-chip had more likely false positives than either calling cards or ChIP-exo, which were comparable to one another (Fig. 5E). The nonresponsive genes that were supported by at least two assays are most likely true bound sites that are nonresponsive for biological reasons. The relatively large size of this set suggests that there are a substantial number of truly bound, nonresponsive genes.

### Combining all available data sets yields the best result

ChIP-exo and calling cards data are not yet available for most yeast TFs. Furthermore, the data sets that are best overall may not be best on every TF. Therefore, we combined the data sets described above using NetProphet 2.0, DTO, and our FDR lower bound. We used the following procedure, which can be applied to any data sets available for any species: Union the network edges produced by performing the following procedure on each perturbation-response data set:

1. Using the entire perturbation-response data set, run a suitable network inference algorithm that does not use binding location data either directly or indirectly. Rank all possible edges according to their score. If desired, multiple inference algorithms can be run (Marbach et al. 2012).
2. For each TF:
  - Compare the network inference scores of the TF's targets to each binding location data set using DTO to select thresholds. Among all binding data sets for the TF, choose the one that yields the best hypergeometric *P*-value.
  - Using the chosen data set and DTO thresholds, check whether the TF is acceptable as defined above. If so, return edges from the TF to targets that are above the thresholds for both expression and binding.

We carried out this procedure on the TFKO and ZEV expression data with the Harbison ChIP, ChIP-exo, and calling cards binding data. For the TFs for which ChIP-exo or calling cards data were available, one of these data sets was chosen over Harbison ChIP 92% of the time (TFKO comparison) or 96% of the time (ZEV comparison). Considering all data sets, the resulting network comprises 96 acceptable TFs with 3268 edges impinging on 1686 unique target genes (Supplemental File S11).

## Discussion

The fundamental question behind this investigation is whether TF binding locations and TF perturbation responses could provide convergent evidence about the direct functional targets of each TF in an organism. Using standard methods to compare binding data from chromatin immunoprecipitation (ChIP) to published perturbation-response data, we found that most of the genes whose *cis*-regulatory DNA is bound by a TF are not functionally regulated by that TF. We found this to be the case for two yeast ChIP data sets as well as ENCODE ChIP-seq experiments in human K562 cells and another 88 ChIP-seq experiments in human HEK293 cells, consistent with previous reports based on different data sets (Hu et al. 2007; Gitter et al. 2009; Lenstra and Holstege 2012; Cusanovich et al. 2014).

If the problem is that most bound genes are not responsive, a natural solution would be to focus on those that are—that is, to take the intersection of the genes a TF binds and the genes that respond to perturbation of the TF as its direct functional targets. However, we proved that this procedure does not effectively identify the direct functional targets when the sets of bound and responsive genes are much larger than their intersection. The reason is that, when there are many genes with nonfunctional binding sites and many genes that respond to the perturbation because they are indirect targets, it is expected that some indirect targets will have nonfunctional binding sites in their *cis*-regulatory DNA. These are not direct functional targets, yet they inhabit and contaminate the intersection of bound and responsive genes.

We quantified this problem by setting minimal criteria for considering the genes that are bound and responsive to be likely direct functional targets. First, the intersection procedure must be able to achieve, in principle, 80% sensitivity with an expected false discovery rate of no more than 20%. Second, the intersection must be larger than would be expected by chance (*P* < 0.01). We say that a TF shows acceptable convergence if it meets both those criteria. This designation does not guarantee that all or most of the TF's bound and responsive genes are responsive because they are bound. The 80–20 criterion is a lower bound on the expected FDR, not an upper bound. Furthermore, it does not guarantee a *unique* relationship between the bound and responsive sets of an acceptable TF—the bound set of one TF can show acceptable convergence when compared to the responsive set of a different TF. *Acceptable* simply means that there is no obvious red flag to prevent us from supposing that a good number of the TF's bound and responsive genes are direct functional targets. When combining ChIP data with steady-state perturbation-response data, the number of TFs showing acceptable convergence was no more than 15% of TFs assayed in both yeast and human data. For the remaining TFs, there is a clear red flag.

We identified four techniques that could substantially increase the number of TFs showing acceptable convergence.

1. Measuring the transcriptional response a short time after inducing overexpression of a TF by using a method such as ZEV.
2. Using dual threshold optimization to set significance thresholds for binding and response data in a way that makes their intersection as significant as possible.
3. Processing all the perturbation-response data together through a network inference algorithm that does not use binding data, either directly or indirectly.
4. Measuring TF binding location by using transposon calling cards or (in yeast) ChIP-exo, rather than standard ChIP.

We combined all these methods to produce a high-quality yeast TF network, using the best binding data available for each TF. Currently, ∼25% of the TFs in the network have binding data from calling cards or ChIP-exo; we expect the network to improve as these data are produced for more TFs. For mammalian cells, calling cards (Wang et al. 2012), dual threshold optimization, and network inference have all been shown to work to some degree. For TF activity perturbation, highly specific genome-targeting systems have been developed and tested with a variety of activation and repression domains (Waryah et al. 2018) and linked to small-molecule inducers (Oakes et al. 2016; Kundert et al. 2019). However, the prospects for obtaining ZEV-like perturbation and calling cards binding data on large numbers of mammalian TFs remain uncertain.

Other new technologies for measuring TF binding locations have shown great promise (Policastro and Zentner 2018) but have not yet yielded a sufficiently large, systematic data set, with matched perturbation-response data, for comparison to ChIP and calling cards. One such technology is DamID, in which a DNA-methyltransferase is tethered to a DNA-binding protein and changes in DNA methylation relative to a control are assayed to determine binding location (van Steensel and Henikoff 2000; Hass et al. 2015; Tosti et al. 2018). Another is CUT&RUN, in which an endonuclease tethered to an antibody against a TF enters permeabilized nuclei and releases the DNA bound by the TF, which diffuses out of the cell and is recovered for sequencing (Skene and Henikoff 2017; Skene et al. 2018; Hainer and Fazzio 2019; Meers et al. 2019). A promising approach for measuring perturbation-response in mammalian cells is to transfect cells with a library of constructs encoding guide-RNAs that target a variety of TFs and then use single-cell RNA-seq to identify the TF perturbed and measure the response. Variants of this general approach include Perturb-seq (Adamson et al. 2016; Dixit et al. 2016; Replogle et al. 2018), CROP-seq (Datlinger et al. 2017), and CRISP-seq (Jaitin et al. 2016). As these technologies mature, they will likely be used to produce large, systematic data sets that can be analyzed using the methods described here.

Even when we apply the best combination of analytic and experimental methods, a large fraction of the genes whose regulatory DNA is significantly bound by a TF does not respond to a perturbation of that TF. Such nonresponsiveness could be caused by several mechanisms.

- Insufficient occupancy—rank response plots (Fig. 5A–C) indicate that the most strongly bound sites are much more likely to be functional than sites that are bound less strongly, even when the weaker sites are statistically significant.
- Saturation—if a gene is already expressed at its maximum possible level and an activator of that gene is induced, no response will be seen. However, if other TFs were removed, lowering the expression level of the gene, it would respond to the induction. The same situation arises when a repressor of an unexpressed gene is induced or an activator of it is depleted.
- Inactivity—the TF may bind DNA even when the TF is in an inactive or partially active state. However, ZEV induction of Gal4 activates galactose genes even in the absence of galactose and presence of glucose, showing that overexpression can elicit a response in conditions where a TF is normally inactive.
- Compensation—the regulatory network as a whole may compensate for the change in TF activity in a way that dampens the effect of the initial perturbation. Measuring responses shortly after the perturbation should reduce the prevalence of such compensation, but some mechanisms can compensate quickly. A simple example would be two essentially equivalent TFs that can bind to the same sites, so that the effects of perturbing one TF are buffered by the other. This was shown to be a contributing factor in a comparison of the Harbison ChIP data to the TFKO data from Hu et al. (2007) (Gitter et al. 2009).
- Override—some regions of a genome may be shut down in a way that overrides the effects of TFs, even when the TFs can bind to the *cis*-regulatory DNA. For example, the transcribed region of a gene might be in inaccessible, tightly compacted DNA even though the *cis*-regulatory region remains somewhat accessible to TFs.
- Synergistic regulation—some TFs that are bound to *cis*-regulatory DNA may be active only where there is a binding site for a cofactor nearby.

Regardless of the mechanism that renders a bound gene nonresponsive, it remains the case that many binding sites are nonfunctional under the conditions tested, in the sense that the transcription rate of the associated gene is unaffected by the presence or absence of the TF. Currently, we do not know how much each of the factors listed above contributes to explaining why so many genes that are bound by a TF do not respond to a perturbation of that TF. For now, technical limitations of the available data sets may be a significant contributing factor. Once those have been mitigated by newer methods like transposon calling cards, we will be in a strong position to investigate the biological factors that explain the nonresponsiveness of genes whose *cis*-regulatory DNA is bound by a TF. Determining the prevalence of each factor will bring the landscape of transcriptional regulation into much clearer focus.

## Methods

Rationale and details can be found in the Online Supplement.

### Data preparation

#### Yeast gene and TF definitions

For all yeast analyses, we considered the 5887 genes labeled “ORF verified” or “uncharacterized” in the *Saccharomyces* Genome Database. The list of 183 TFs is in Supplemental File S9.

#### Yeast binding location data sets

We downloaded the *P*-values for the Harbison et al. (2004) data from http://younglab.wi.mit.edu/regulatory_code/GWLD.html. Following the authors’ recommendation, targets with *P* ≤ 0.001 were considered significantly bound. We downloaded the occupancy-level profiles for Venters et al. (2011) from their Supplemental Table S4a. The log2 fold change of experimental signal over background signal within each promoter was used as the binding signal strength. The “25&37C merged MockIP controls” file was obtained from the authors. ChIP-exo data for 26 TFs were compiled from several studies (Rhee and Pugh 2011; Bergenholm et al. 2018; Rossi et al. 2018a,b; Holland et al. 2019). We obtained data directly from the authors of Holland et al. (2019) and Bergenholm et al. (2018) and focused on the glucose-limited chemostat data as that gave the best agreement with both TFKO and ZEV15 response data (Supplemental Fig. S5). We combined calling cards data from Wang et al. (2011) and Shively et al. (2019) on Cbf1, Cst6, Gal4, Gcr1, Gcr2, Rgm1, and Tye7 with new data on Eds1, Gcn4, Ino4, Leu3, Lys14, Rgt1, Sfp1, and Zap1 (Supplemental File S10).

#### Yeast perturbation-response data

The TFKO data (Kemmeren et al. 2014) are from http://deleteome.holstegelab.nl/data/downloads/deleteome_all_mutants_controls.txt. The ZEV induction system is described in Hackett et al. (2020). We used the column log2_shrunken_timecourses from the file “Raw & processed gene expression data” at https://idea.research.calicolabs.com/data. Genes with nonzero entries were considered responsive.

#### Human ChIP data

All ENCODE data were downloaded from https://www.encodeproject.org on 1/21/19. We used the “conservative” ChIP-seq peaks called by the ENCODE pipeline (Irreproducible Discovery Rate ≤ 2%). The HEK293 data file (Schmitges et al. 2016) was downloaded from NCBI Gene Expression Omnibus (GEO; https:// www.ncbi.nlm.nih.gov/geo/) series GSE76494 (file GSE76494_ combined_summits .motif_hits.per_protein.hg19.tar.gz). We defined the regulatory region of a gene as a core promoter (TSS ± 500 bp) combined with the gene's “double elite” enhancers from GeneHancer V4.8 (Fishilevich et al. 2017), which we obtained from the authors. To quantify each TF-target binding interaction, we summed the log_10_ *Q*-values of significant peaks (ENCODE) or the scores of all summits (HEK293) within the target's regulatory regions.

#### Human perturbation-response data

ENCODE: Differentially expressed genes in each strain were assessed with DESeq2 (V1.10.1) (Love et al. 2014). HEK293 RNA-seq data were downloaded from NCBI GEO Series GSE76495 (file GSE76495_OE .vsd_normalized.log2.txt.gz). Since there were no control replicates, we used the expression levels in each profile, normalized to the medians of the respective batches, as the response strength (Schmitges et al. 2016).

### TF network mapping

All software was run with default parameters. NetProphet 2.0 (Kang et al. 2018) was downloaded from https://github.com/yiming-kang/NetProphet_2.0; GENIE3 (Huynh-Thu et al. 2010) (v1.16.5, Python implementation) from http://www.montefiore.ulg.ac.be/~huynh-thu/software.html; Inferelator (Greenfield et al. 2013) from https://github.com/ChristophH/Inferelator; and MERLIN (Roy et al. 2013) from https://github.com/marbach/gpdream. No prior network was used with Inferelator because our intention is to infer networks without any influence from binding data.

### Dual threshold optimization

#### DTO algorithm

DTO can be used to compare any two ranked lists of genes. A series of threshold pairs, one for each list, are tried and genes above the threshold are considered “positives” in the corresponding list. The series of thresholds for each ranked list, *T*_1_, *T*_2_, …, was generated by using the recurrence: *T*_1_ = 1; $T_{n}\;=\;Floor(T_{n-1}*1.01\;+\;1)$. This produces a finer spacing among higher ranks. For each pair of thresholds (one for each ranked list), a hypergeometric *P*-value for overlap of the positive gene sets is computed. DTO returns the threshold combination that minimizes this *nominal P-value*. A null distribution for testing the significance of the overlap chosen by DTO was generated from the nominal *P*-values chosen in 1000 runs of DTO on 1000 randomized rankings.

#### Application of DTO to yeast data

The universe was defined as the set of all genes assayed in both of the two data sets being compared. Genes were ranked according to *P*-values for data sets that had them, or log fold change (ZEV data, column log2_cleaned_ratio from the file “Raw & processed gene expression data” at https://idea.research.calicolabs.com/data) or score (NetProphet 2.0, GENIE3, Inferelator, and MERLIN).

#### Application of DTO to human data

For all perturbation data sets, the universe for hypergeometric probabilities was the set of all genes detected in the data set. For each perturbed TF in ENCODE data, the “RSEM expected counts” in the perturbation samples were compared to those from the control set using DESeq2 (V1.10.1). The genes were ranked by the differential expression *P*-values. DTO was limited to choosing bound or responsive genes with *P* ≤ 0.1. When applied to network inference scores, it was limited to the top 500,000 edge scores. For HEK293 data, genes are ranked by the absolute value of the log fold change relative to control samples, as no replicates or *P*-values were available for most TFs. For both raw perturbation data and network inference scores, the score of the TF-target relationship was required to be among the top 300,000 scores.

### Rank-response plots

A gene was considered responsive in the TFKO data if it had an adjusted *P* < 0.05 and in the ZEV15 data if it had an absolute shrunken log fold change >0. For each TF, let *n* be the smaller of the numbers of responsive genes in the TFKO and ZEV15 data. We labeled the top *n* most strongly responsive genes in the TFKO and ZEV15 data as responsive for purposes of Fig. 5A–C. This equalized the number of ZEV15-responsive and TFKO-responsive genes for each TF. We then sorted genes by the strength of their binding signal for the TF and considered the top 1, 2, 3, 4, etc. most strongly bound genes. For each such group, we plotted the fraction of genes that were responsive.

### GO enrichment analysis

Gene Ontology biological process terms were obtained from R Bioconductor library org.Sc.sgd.db (V3.5.0) and terms annotated to less than three or >300 genes were eliminated. If multiple terms were enriched by the same set of targets, only the most specific term was retained. Term enrichment for the targets of each TF was analyzed using the hypergeometric test (GOstats V2.44.0). In Fig. 4D, enrichment was calculated separately for each network. The most significant term is shown, regardless of the network from which it was obtained.

## Data access

All raw and processed sequencing data generated for this study using transposon calling cards have been submitted to NCBI Gene Expression Omnibus (GEO; https://www.ncbi.nlm.nih.gov/geo/) under accession number GSE144657. Software implementing dual threshold optimization and instructions is available at GitHub (https://github.com/BrentLab/Dual_Threshold_Optimization) and as Supplemental Code.

## Competing interest statement

R.S.M., B.J.W., and G.K. are employees of Calico Life Sciences LLC. Other authors declare that they have no competing interests.

## Supplementary Material

Supplemental Material
