# Supplementary material for: Dual threshold optimization and network inference reveal convergent evidence from TF binding locations and TF perturbation responses
Source: Genome Res. 2020 Mar;30(3):459–71. doi: 10.1101/gr.259655.119 (PMC7111528; doi:10.1101/gr.259655.119)
Supplement: Supplemental Material [file supp_30_3_459__index.html]

Dual threshold optimization and network inference reveal convergent evidence from TF binding locations and TF perturbation responses — Supplemental Material 

# Dual threshold optimization and network inference reveal convergent evidence from TF binding locations and TF perturbation responses

## Supplemental Material

- Supplemental\_Materials.pdf
- Supplemental\_File\_S1.xlsx
- Supplemental\_File\_S2.xlsx
- Supplemental\_File\_S3.xlsx
- Supplemental\_File\_S4.xlsx
- Supplemental\_File\_S5.xlsx
- Supplemental\_File\_S6.xlsx
- Supplemental\_File\_S7.xlsx
- Supplemental\_File\_S8.xlsx
- Supplemental\_File\_S9.xlsx
- Supplemental\_File\_S10.tar.gz
- Supplemental\_File\_S11.xlsx
- Supplemental\_Code.tar.gz
