# Supplementary material for: Dual threshold optimization and network inference reveal convergent evidence from TF binding locations and TF perturbation responses
Source: Genome Res. 2020 Mar;30(3):459–71. doi: 10.1101/gr.259655.119 (PMC7111528; doi:10.1101/gr.259655.119)
Supplement: Supplemental Material [file supp_gr.259655.119_Supplemental_Materials.pdf]

SUPPLEMENTAL BOX AND FIGURES

Box S1. Expected False Discovery Rate (FDR) of intersection algorithms

Intersection algorithms identify the direct functional targets of a TF as those whose promoters are bound by the TF in an assay such as ChIP-Seq and are responsive when the same TF is perturbed. A true direct functional (DF) target is responsive when the TF is perturbed *because it is bound by the TF*. One possible alternative is that a gene is in the intersection because it is an indirect target of a TF and happens, by chance, to have a non-functional binding site for the same TF in its promoter. Although we use the terms non-functional binding site and indirect target, the analysis is unaffected if these are simply false positives of the binding or response assays.

We start by defining the following notation for any given TF:

B	the set of genes whose promoters appear to be bound by the TF in an experiment
R	the set of genes that appear to be responsive when the TF is perturbed in an experiment
G	the set of all genes assayed in both the binding and response experiments
DF	the true set of direct functional targets of the TF

DF is unknown, but B, R, and G are all observed outcomes of the experiments. $B \cap \overline{DF}$ is the set of genes with only non-functional binding of the TF (the overbar indicates set complement). Genes with functional binding are in $B \cap DF$. Likewise, $R \cap \overline{DF}$ is the set of indirect targets – genes that are responsive but not direct functional targets.

This analysis is based on the idea that the promoters with only non-functional binding for a TF can be modeled as though they were scattered randomly across genes, without regard to whether the genes are indirect targets of the same TF. (In fact, we only need to assume that promoters with non-functional binding don't systematically avoid indirect target genes.) We believe this is a good assumption because we cannot think of any molecular or evolutionary mechanism by which non-functional binding sites could be enriched or depleted in the promoters of indirect target genes. Since they are non-functional, they are not under any evolutionary selection. The same applies to false positives of the binding and/or response assays – there is no reason to believe that false positives of the binding assay would be enriched or depleted among the false positives of the response assay.

According to this model, the genes with only non-functional binding are selected at random from $\overline{DF}$, so the expected fraction that are also in $R \cap \overline{DF}$ is simply $|R \cap \overline{DF}|/|\overline{DF}|$, where the vertical bars indicate set size. Thus

$$E[|R \cap B \cap \overline{DF}|] = |B \cap \overline{DF}| \frac{|R \cap \overline{DF}|}{|\overline{DF}|}$$

By way of analogy, it is as though $|B \cap \overline{DF}|$ balls were selected at random from a jar containing $|\overline{DF}|$ balls, of which $|R \cap \overline{DF}|$ are red and the remainder are white. The expected number of red balls is given by the right hand side of formula above.

By definition, the false discovery rate of the intersection algorithm is:

$$FDR = \frac{|R \cap B \cap \overline{DF}|}{|R \cap B|},$$

The denominator is directly observable from the assays. We do not know the true set of DF targets, but have just derived the expectation of the numerator with respect to the random process that distributes promoters with non-functional binding sites to genes.

We also observe that the sensitivity of the intersection algorithm is, by definition:

$$\text{Sn} = \frac{|DF \cap R \cap B|}{|DF|} \leq \frac{|R \cap B|}{|DF|}$$

So

$$|DF| \leq \frac{|R \cap B|}{\text{Sn}} \tag{1}$$

Putting these equations together,

$$
\begin{aligned}
E[FDR] &= \frac{E[|R \cap B \cap \overline{DF}|]}{|R \cap B|} \\
&= \frac{|B \cap \overline{DF}||R \cap \overline{DF}|}{|\overline{DF}||B \cap R|} \\
&\geq \frac{\max(0, |B| - |DF|)\max(0, |R| - |DF|)}{|\overline{DF}||B \cap R|} \\
&\geq \frac{\max(0, |B| - |R \cap B|/\mathsf{Sn})\max(0, |R| - |R \cap B|/\mathsf{Sn})}{|\overline{DF}||B \cap R|} \\
&\geq \frac{\max(0, |B| - |R \cap B|/\mathsf{Sn})\max(0, |R| - |R \cap B|/\mathsf{Sn})}{|G||B \cap R|}
\end{aligned}
$$

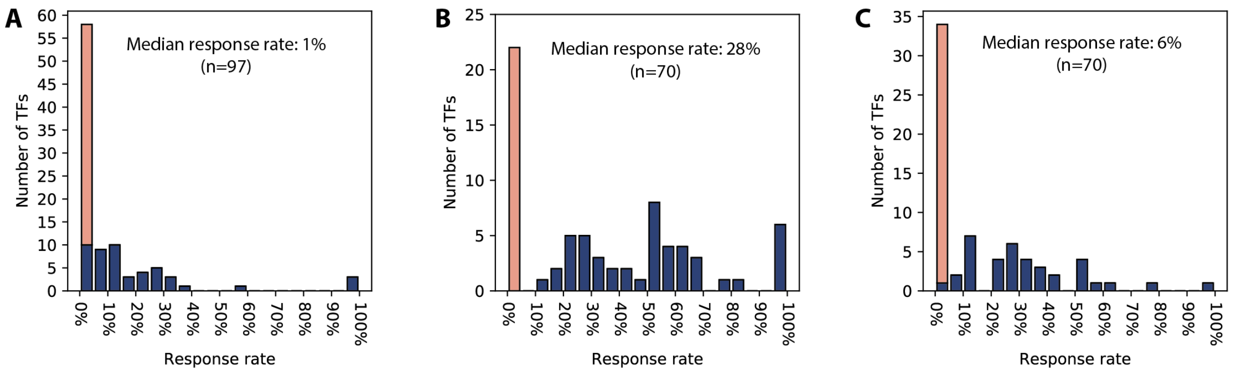

Figure S1. Same as Fig. 1A except: (A) Binding threshold is p<0.001 and response threshold is p<0.05 with fold change > 1.5. Total bound and responsive genes is 209. (B) Binding threshold is p<0.00001 and response threshold is p<0.05 with no minimum fold change. Total bound and responsive genes is 297. (C) Binding threshold is p<0.00001 and response threshold is p<0.05 with fold change > 1.5. Total number of bound and responsive genes is 119.

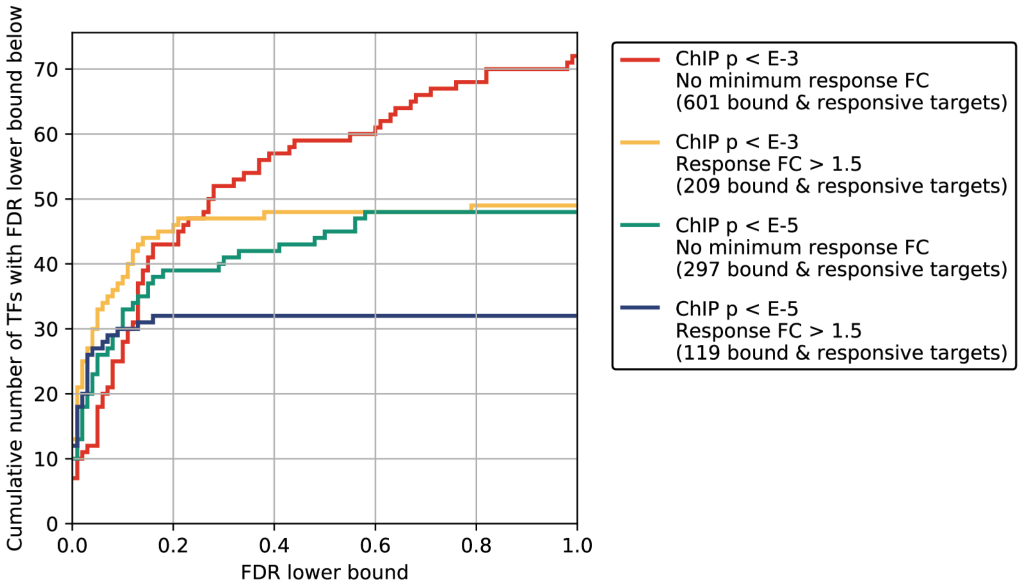

Figure S2. Cumulative number of TFs with expected FDR lower bound less than the number on the horizontal axis, assuming sensitivity of 80% (see Box S1). Red line: moderate binding and response thresholds; orange line: moderate binding threshold and tight response threshold; green line: tight binding threshold and moderate response threshold; blue line: tight binding and response thresholds.

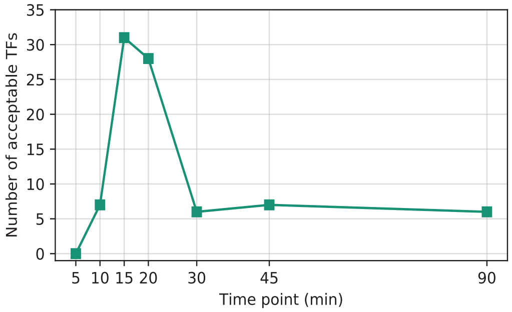

Figure S3. Numbers of acceptable TFs when comparing Harbison ChIP data to ZEV response data at various time points.

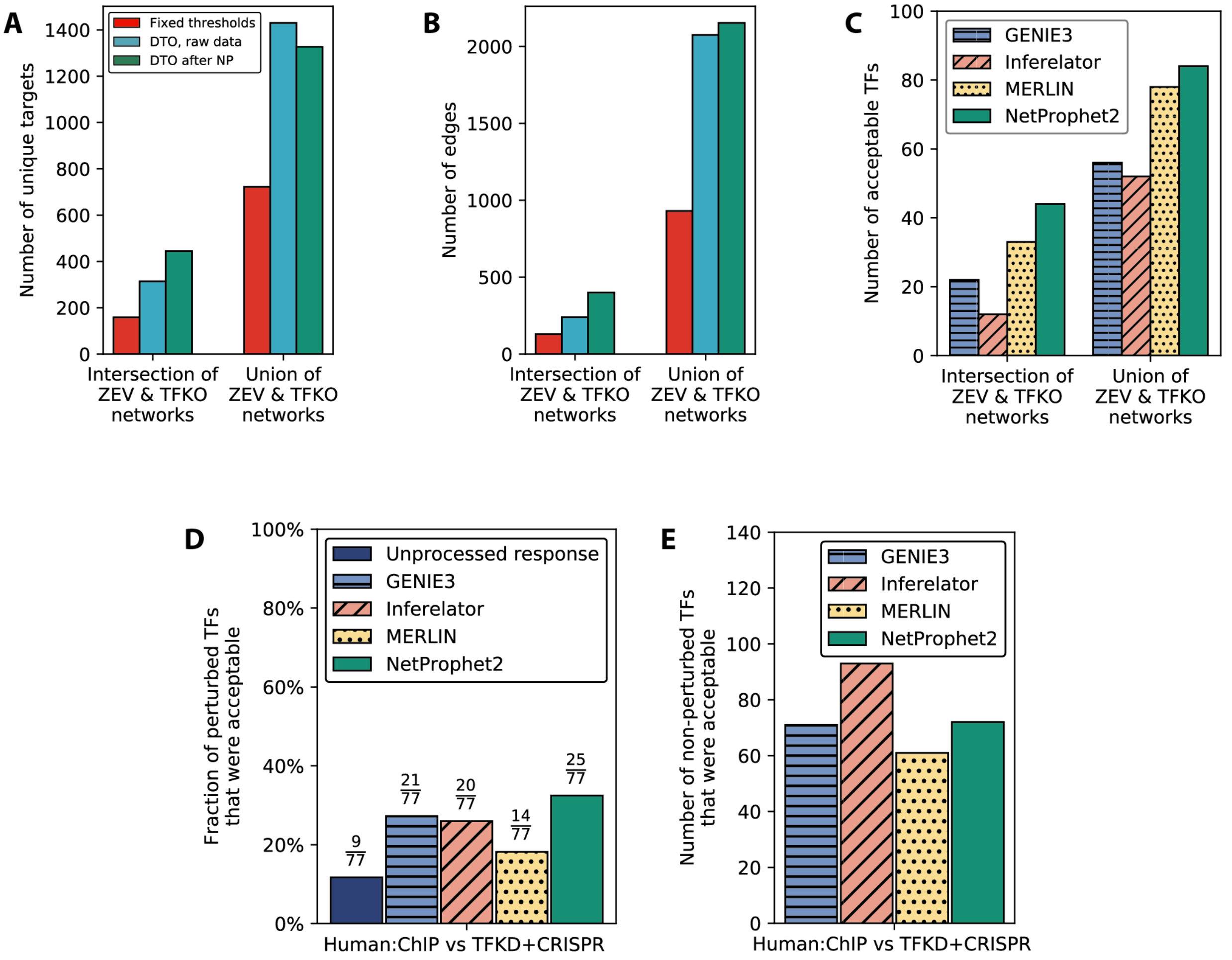

Figure S4. (A) and (B) Comparison of TFKO and ZEV15 networks derived from fixed thresholds, dual threshold optimization (DTO) on raw gene expression, and DTO on gene expression data processed by NetProphet 2.0. DTO on the raw expression data (blue bars) increases the size of the networks over fixed thresholds (red bars). This is true for both the intersection of the TFKO and ZEV networks (left bar grouping) and their union (right bar grouping). Post processing expression data with NetProphet 2.0 (green bars) further increases the size of the networks. See Fig. 2 for the numbers of TFs showing acceptable convergence in these analyses. (C) Comparison of yeast networks derived from DTO on ChIP data and response data processed using several network inference algorithms (D) Comparison of human K562 networks derived from DTO on ChIP-seq and raw response data or network inference processed response data. Bar height is the fraction of TFs showing acceptable convergence divided by the number of TFs that were ChIPped and perturbed by either TFKD or CRISPR. Network inference postprocessing improves convergence over unprocessed response data. (E) Similar to (D), but considering only TFs that were not directly perturbed. These TFs cannot be analyzed for convergence without network inference.

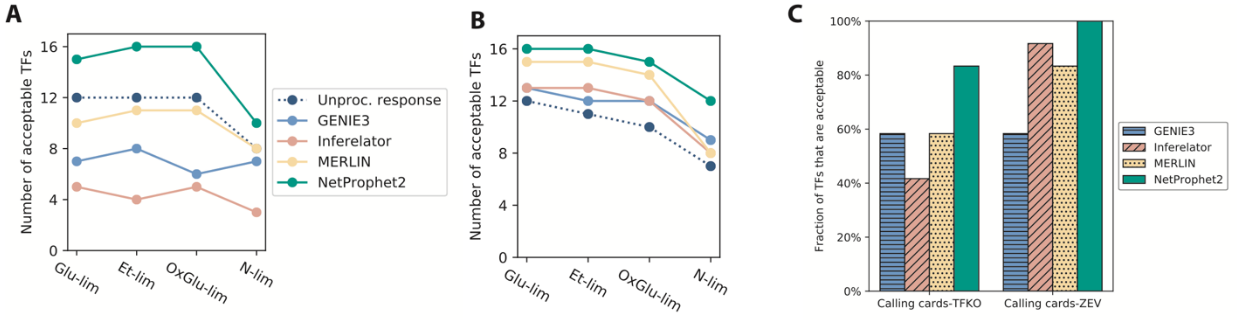

Figure S5. (A) Among 16 TFs for which we have data in TFKO and ChIP-exo in four nutrient limited conditions, the number of TFs that show convergence between ChIP-exo data and TFKO response data (dotted blue) or network inference processed response data (other colored lines). (B) Same as (A) except that ZEV15 data replaces TFKO data. (C) Comparison of networks derived from DTO on calling cards data and response data processed through several network inference algorithms.

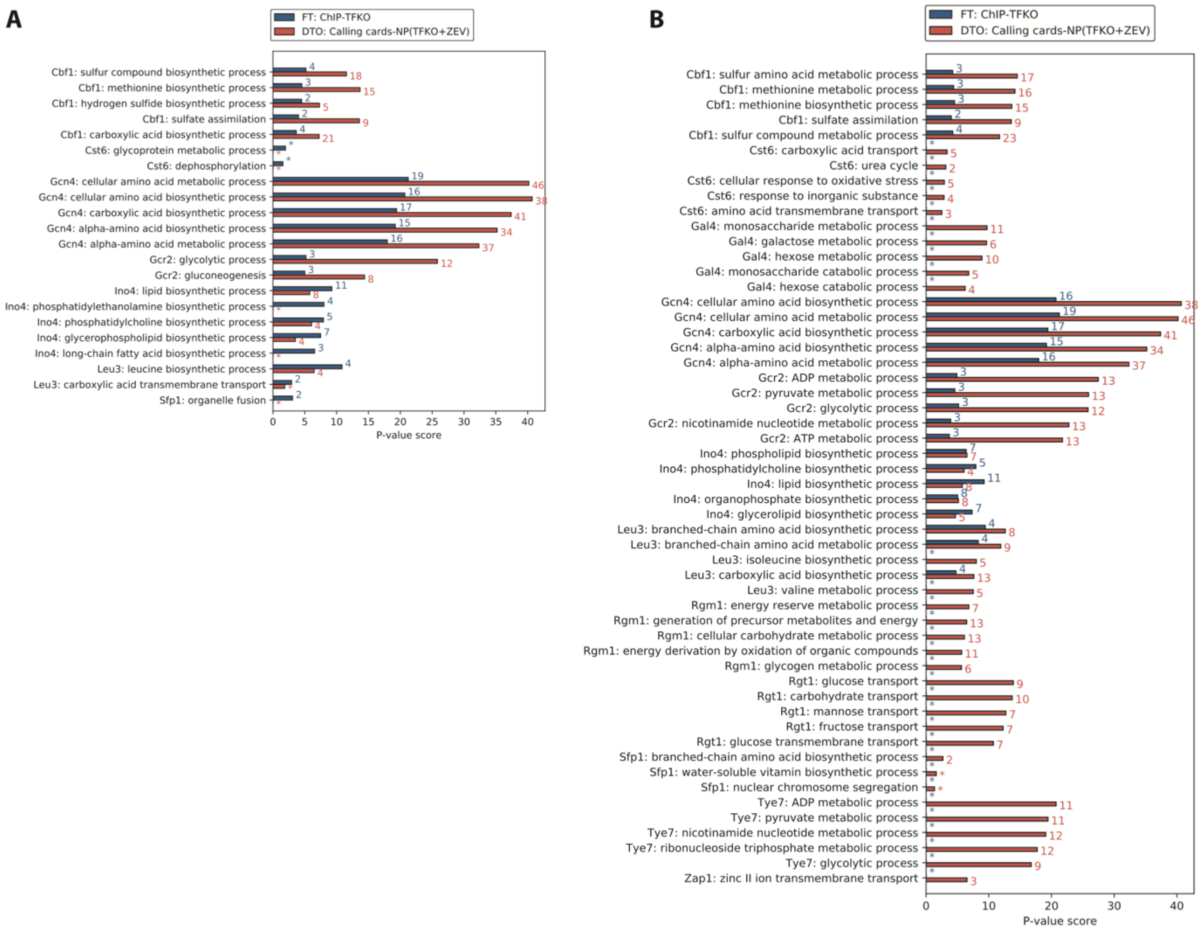

Figure S6. Same as Fig. 4D except: (A) For each TF that has any enriched GO term, the five most enriched terms when the targets of each TF are chosen by using fixed thresholds on Harbison ChIP and TFKO data. In most cases these same terms are even more significantly enriched when the targets are chosen by using a different method -- dual threshold optimization comparing calling cards data to output from NetProphet 2.0 run on the TFKO and ZEV expression data (red bars). The numbers to the right of the bars indicate the number of genes with a given GO term among the targets of the TF. (B) For each TF that has any enriched GO term, the five most significantly enriched terms chosen by using dual threshold optimization comparing calling cards data to output from NetProphet 2.0 run on the TFKO and ZEV expression data. In many cases, terms with one or two fewer genes are more familiar than the terms with the most genes. For example, Gcr2 has 12 target genes annotated with “glycolytic process”, corresponding to its accepted function, but the most significant term is “ADP metabolic process”, which contains those 12 glycolytic genes plus one additional gene.

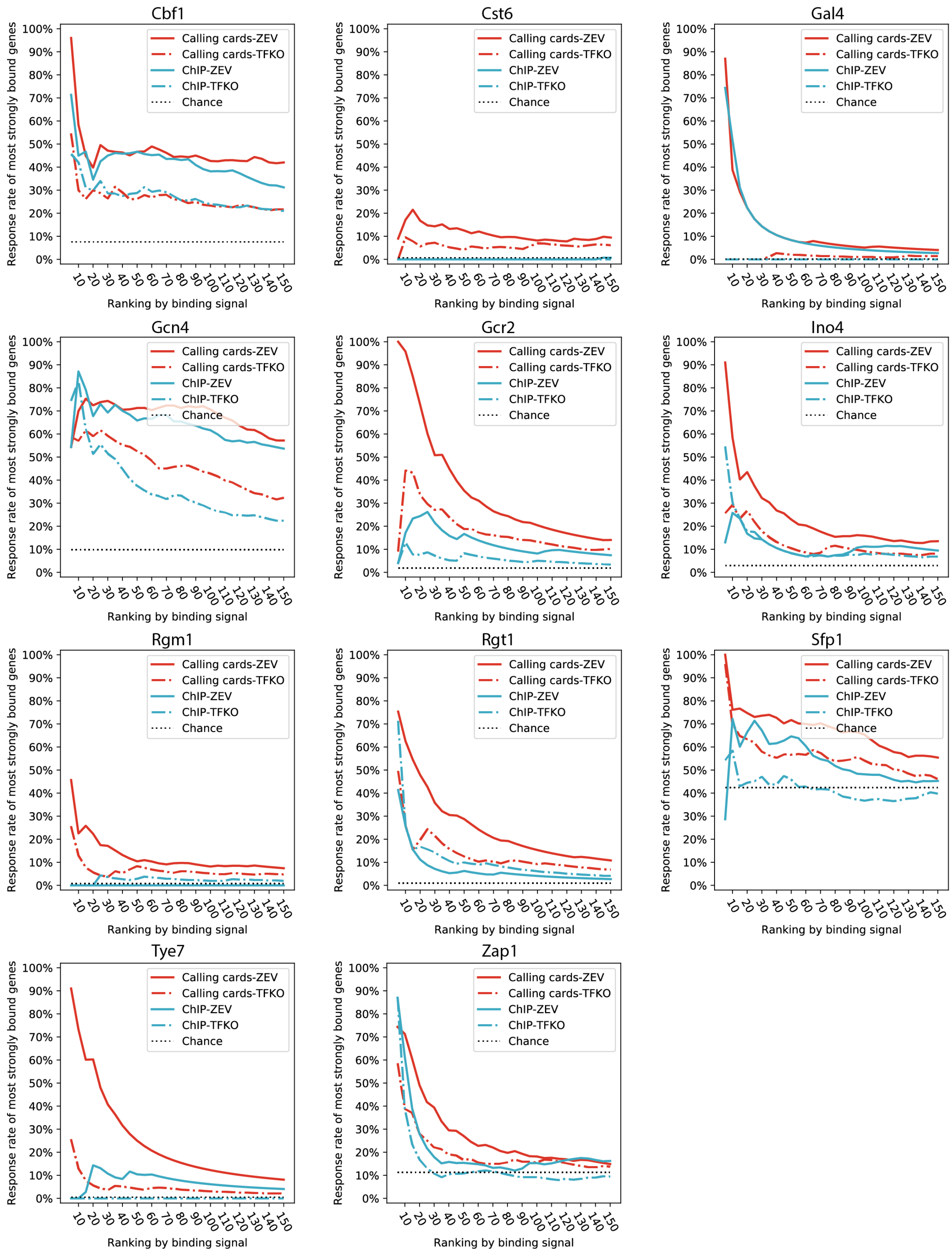

Cbf1
Cst6
Gal4
Gcn4
Gcr2
Ino4
Rgm1
Rgt1
Sfp1
Tye7
Zap1
Calling cards-ZEV
Calling cards-TFKO
ChIP-ZEV
ChIP-TFKO
Chance
Response rate of most strongly bound genes
Ranking by binding signal
0%
10%
20%
30%
40%
50%
60%
70%
80%
90%
100%
10 20 30 40 50 60 70 80 90 100 110 120 130 140 150

Figure S7. Same as Fig. 5A. The fraction of most strongly TF-bound genes that are responsive to the perturbation of that TF, as a function of the number of most-strongly bound genes considered. Shown are the other 11 TFs, in addition to Leu3, that had data available in Harbison ChIP, transposon calling cards, TFKO, and ZEV15.

SUPPLEMENTAL METHODS

DATA PREPARATION

Yeast gene and TF definitions

For all yeast analyses, we considered the 5,887 genes labeled as "ORF verified" or "uncharacterized" in the Saccharomyces Genome Database (SGD), discarding the 1,127 labeled as "dubious", "ncRNA", "rRNA", "snoRNA", "snRNA", or "tRNA". We only considered TFs with evidence of direct DNA binding via a DNA binding domain. To identify these, we compared multiple lists, including those that had been ChIPped by Harbison et al., those that were over-expressed in the ZEV data, and those that had DNA binding specificity models in the CIS-BP database (Weirauch et al. 2014). In cases of disagreement, we curated the list manually by consulting data in SGD, focusing primarily on domain analysis of the protein and on gene ontology categories assigned via high-throughput experiments such as protein-binding microarrays. In most cases the judgment is clear but there are some borderline cases that require a best guess. The complete list of 183 yeast proteins that we treated as TFs is provided as Supplemental File S9.

Yeast ChIP-chip data sets

The Harbison ChIP-chip binding location data was published in (Harbison et al. 2004). We downloaded the p-values that represent the significance of TF binding within the intergenic regions from http://younglab.wi.mit.edu/regulatory_code/GWLD.html. Following the authors' recommendation, targets were considered significantly bound if their p-value was less than or equal to 0.001. TFs with no significantly bound targets were eliminated from further analysis. The Venters ChIP-chip data were published in (Venters et al. 2011). We downloaded the occupancy-level profiles for 200 transcription-related proteins from Table S4a in (Venters, et al. 2011). The log2 fold change of experimental signal over background signal within each promoter was used as the binding signal strength. The probes covered a distal region (260-320 bp upstream of ATG) and a proximal region (30-90 bp upstream of ATG). The downloaded occupancy level took the maximal level from either regulatory region. The authors used an FDR threshold of 5%, but we used 1% in order to make the data more comparable to those from the Harbison data set. For each TF, an FDR cutoff was calculated by searching for an occupancy level such that the ratio of number of targets in the mock IP control over the number in the experimental sample reaches the desired FDR. The "25&37C merged MockIP controls" file was obtained directly from the authors as it was unpublished. TFs with no significantly bound target were eliminated from further analysis.

Yeast ChIP-exo data sets

The ChIP-exo data for 26 TFs were compiled from four resources (Bergenholm et al. 2018; Holland et al. 2019; Rhee and Pugh 2011; Rossi et al. 2018a, 2018b). We downloaded genomic coordinates of the ChIP peaks for Reb1, Gal4, Phd1 and Rap1 published in ref. (Rhee and Pugh 2011) from https://ars.els-cdn.com/content/image/1-s2.0-S0092867411013511-mmc2.xls. We mapped the peaks to genes using the coordinates of each gene's promoter region (700 bp upstream to ATG) in reference genome S288C-R55, which was the last release prior to the date cited in the paper, "(build: 19-Jan-2007)" (ref. (Engel et al. 2014) lists all releases). We then

calculated each TF's binding strength at each promoter as the sum of all the TF's in that promoter. We downloaded peaks for Abf1 and Ume6 generated using a newer protocol, ChIP-exo 5.0, described in ref. (Rossi, et al. 2018b), from GEO Series GSE110681. We also downloaded peaks for Cbf1 from GEO Series GSE93662 (see ref. (Rossi, et al. 2018a)). The assignment of peak-promoter and binding strength at promoter were calculated as for the data from Rhee, et al., 2011, except that both peak and promoter coordinates were based on gene annotation from reference genome S288C-R64. Lastly, we obtained ChIP-exo data for 20 TFs (Cat8, Cbf1, Ert1, Gcn4, Gcr1, Gcr2, Hap1, Ino2, Ino4, Leu3, Oaf1, Pip2, Rds2, Rgt1, Rtg1, Rtg3, Sip4, Stb5, Sut1, Tye7) directly from the authors of (Holland, et al. 2019) and (Bergenholm, et al. 2018). Each TF was assayed in four different environmental conditions, but we focused on the glucose limited chemostat data as that gave the best agreement with both TFKO and ZEV15 response data. This data set contains scores for each promoter that had at least one peak assigned to that promoter. We directly used the highest score at each promoter as the binding strength, after removing any peak that was > 700 bp upstream from ATG. Any promoter without a score in the file was assigned a score of zero.

Yeast transposon calling cards data

We combined calling cards data from (Wang et al. 2011) and (Shively et al. 2019) on Cbf1, Cst6, Gal4, Gcr1, Gcr2, Rgm1, and Tye7 with new data on Eds1, Gcn4, Ino4, Leu3, Lys14, Rgt1, Sfp1, and Zap1 (File S10). For ease of access, we provide P-values for promoter binding from the combined data sets as Supplemental File S10. For each TF, all data from all replicates were combined. Transpositions within the promoters of yeast genes (700 bp upstream to ATG, reference genome S288C-R61) were used for calculating the significance of TF binding. For each promoter, a Poisson p-value was calculated by comparing the experiment sample with a no-TF control sample as described (Wang et al. 2012). To obtain a ranking by calling cards signal strength for dual threshold optimization, we used the normalized transposition count of the experimental samples minus that of the control samples to break ties when promoters had identical p-values.

Yeast TFKO data

The microarray expression data on gene knockout strains was published in ref (Kemmeren et al. 2014). The gene expression profiles of 1,484 single gene deletion strains and 3 wild type replicates were downloaded from http://deleteome.holstegelab.nl/data/downloads/deleteome_all_mutants_controls.txt. In addition, we downloaded the gene expression profiles after removal of the slow growth signature removed http://deleteome.holstegelab.nl/data/downloads/deleteome_all_mutants_svd_transformed.txt. This transformed data set did not contain new p-values and analyzing it did not produce better results than the untransformed data, so we focused on the untransformed data.

Yeast ZEV induction data

The ZEV induction system was described in ref. (Hackett et al. 2019). The shrunken expression profiles were used as the quantitative responsiveness of target genes after TF induction. Specifically, the file "Raw & processed gene expression data" was downloaded from

https://idea.research.calicolabs.com/data and the column labeled `log2_shrunken_timecourses` was used. The responsive set contains all targets with non-zero expression levels. We systematically analyzed ZEV expression profiles measured at all time points (5, 10, 15, 20, 30, 45, and 90 minutes) after TF induction. To make different time points comparable, we only focused on 103 TFs that were available in the Harbison ChIP-chip data and each time point of the ZEV data. The maximal number of acceptable TFs was obtained at 15 min, so we chose to move forward with this time point for all subsequent analyses except those that involve network inference. For network inference, we used the 15, 45, and 90 minute samples.

Human ChIP-seq data

Two human ChIP-seq data sets were analyzed in this work: ChIP-seq in K562 cell line published by ENCODE, and ChIP-seq in HEK293 cell line published in ref. (Schmitges et al. 2016). All ENCODE data were downloaded from www.encodeproject.org as of January 21st, 2019. We focused on the data on K562 cells because it had by far the most TFs with both ChIP-seq and perturbation response data. We downloaded the "conservative" ChIP-seq peaks mapped to GRCh38 as called by the ENCODE pipeline, which uses the Irreproducible Discovery Rate (IDR) analysis of biological replicates with 2% IDR cutoff. Using the ENCODE definition of transcription factor, there was ChIP-seq data for 261 TFs in K562. To quantify the significance of each TF-target binding interaction, we summed the log10 q-values of significant peaks that were within the regulatory regions of each gene (defined below). For the HEK293 cell line, ChIP-seq was carried out using an antibody against GFP. We downloaded the combined summits for 131 ChIPped zinc finger proteins from GEO Series GSE76494 (see ref. (Schmitges, et al. 2016) for details). The binding strength within the regulatory regions of a gene was the summed scores of all summits assigned to those regions. We tried two definitions of regulatory region: (1) a single long promoter extending from 10 Kb upstream of the 5'-most transcription start site (TSS) to 2Kb downstream (Ensembl Release 92), or (2) a core promoter extending from 500 bp upstream of the TSS to 500 bp downstream combined with the gene's enhancers from the GeneHancer database V4.8 (Fishilevich et al. 2017). We used only the "double elite" enhancers, for which both the existence of the enhancer and the gene-enhancer association are supported by at least two evidence sources. This double-elite list was obtained by emailing the authors of the paper. In order to properly use the ChIP summits in HEK293 whose coordinates were based on GRCh37, we used the LiftOver tool in UCSC genome browser to lift over the coordinates of regulatory regions from GRCh38 to GRCh37.

Human ChIP-exo data

A collection of ChIP-exo data of 221 KRAB zinc-finger proteins in HEK293T cell lines was downloaded from GSE78099 (Imbeault et al. 2017). We mapped the MACS peaks obtained from the supplemental files to the regulatory regions defined above (GRCh37). We then summed the scores of peaks for each gene to represent the binding strength between each protein and its target.

Human TFKD, CRISPRi and TF-induction data

We considered three human perturbation response data sets: TF knockdown (TFKD) in K562,

CRISPRi in K562, and TF-induction in HEK293 (Schmitges, et al. 2016). The RNA-seq expression profiles of wild-type controls, TFKDs and CRISPRi were downloaded from the ENCODE web site. Knockdowns using small-interfering RNA (siRNA) or small-hairpin RNA (shRNA) were combined in the data set we referred to as TFKD while the CRISPRi and CRISPR TF-disablement data were combined in the data set we referred to as CRISPR. For K562 cells, there were TFKD experiments targeting 261 different proteins and CRISPRi experiments targeting 96 different proteins. The expected counts were reported by the RSEM program in the ENCODE RNA-seq processing pipeline using gene annotation from GENCODE V24 (GRCh38). Differentially expressed genes in each perturbed TF strain were processed by comparing the experimental replicate set to the control set using DESeq2 (V1.10.1). On the TF-induction for HEK293, RNA-seq was carried out 24 hours after overexpressing the TF from a tetracycline-inducible plasmid. For the majority of TFs there was only a single replicate of the RNA-seq experiment, which prevents the calculation of statistical significance by traditional methods. The processed RNA-seq expression profiles (after lowly-expressed gene removal and batch normalization) for 80 induced zinc finger proteins were downloaded from GEO Series GSE76495. Since there were no control replicates, we used the expression levels in each profile, normalized to the medians of the respective batches, as the response strength (Schmitges, et al. 2016).

NETPROPHET ANALYSIS

NetProphet 2.0 is a TF network inference algorithm that exploits gene expression data under genetic or environmental perturbation and genome sequences with annotations. The algorithm is described in detail in ref. (Kang et al. 2017). Here, two yeast TF networks were mapped, one using the Kemmeren TFKO gene expression data and one using the ZEV data. Three human TF networks were mapped using the three perturbation response data sets.

<u>Yeast NP networks</u>

NetProphet 2.0 requires gene expression data in the form of a gene expression matrix and differential expression matrix. The Kemmeren gene expression matrix was represented as the log2 fold-change (logFC) values of strains with gene deletions over wild-type strains. The ZEV gene expression matrix was represented as the logFC values of the levels measured at a certain time point after the TF induction relative to time 0. For the differential expression (DE) module of the algorithm, we used Kemmeren samples in which a TF-encoding gene was knocked out, not those in which some other type of gene was knocked out, and ZEV samples from 15 min after TF induction. For the co-expression module of the algorithm, we used the complete set of 1,484 Kemmeren expression profiles from strains lacking one gene (not necessarily encoding a TF) or 590 ZEV expression profiles from 15 minutes, 45 minutes, or 90 minutes post-induction. The other two inputs were DNA sequences of yeast promoters and amino acid sequences of TFs' DNA binding domains (DBDs), as described in ref. (Kang, et al. 2017). PWM models of TFs' binding specificity were <u>not</u> used. Each output network is an adjacency matrix, where the rows represent TFs, the columns represent genes, and the entries are NetProphet scores representing the aggregate strength of evidence that the gene is a direct functional target of the TF.

Human NP networks

For K562 data, we calculated differential expression (DE) p-values for TFKD and CRISPRi independently using DESeq2. The DE matrix input to NetProphet 2.0 contained the -log p-values, with a negative sign for apparent repression (the knockdown of the TF makes the target gene go up). For HEK293 data, we directly used the logFC values because there were no replicates, hence no p-values, for most TFs. The co-expression matrix contained the logFC of individual mutant strain replicates over the median expression level of control replicates (K562) or the median expression level of the gene across all perturbations (HEK293). There were 765 expression profiles (including replicates) for K562 TFKD, 252 for K562 CRISPRi, and 107 for HEK293 TF inductions. We obtained DNA sequences of the regulatory regions based on their coordinates in GRCh38 using our definition (2) of regulatory regions. We concatenated the enhancers and promoter of each gene into a single sequence for the purpose of motif inference in NetProphet 2.0. Each pair of concatenated regions was separated by 50 N's to ensure that no inferred motif instances crossed between one enhancer and another. We also queried the CIS-BP (Weirauch, et al. 2014) database for the amino acid sequences of human TFs' DBDs. The details of DBD preprocessing are described in (Kang, et al. 2017). The TFKD and CRISPRi networks had 392 TFs each, which were ENCODE TFs being ChIPped in either of the major cell lines K562 or HepG2. The TF-induction network had 103 TFs, which were the zinc finger proteins being ChIPped in HEK293.

ANALYSIS USING OTHER NETWORK INFERENCE ALGORITHMS

GENIE3 (Huynh-Thu et al. 2010) (v1.16.5, Python implementation) was downloaded from http://www.montefiore.ulg.ac.be/?huynh-thu/software.html. Default parameters in GENIE3 were used. The version of Inferelator that incorporates Bayesian Best Subset Regression (Greenfield et al. 2013) was downloaded from https://github.com/ChristophH/Inferelator. No prior network was used because our intention is to infer networks from perturbation response data without any influence from data on TF binding locations. Otherwise, default parameters were used. MERLIN (Roy et al. 2013) was downloaded from https://github.com/marbach/gpdream and used with default parameters. We input the same gene expression matrix, TF list and gene list to NetProphet 2.0, GENIE3, Inferelator, and MERLIN.

ACCEPTABLE TFS

For each pair of binding and expression data on a given TF, the positive gene sets (bound genes or responsive genes) were compared. The TF was deemed acceptable if they met two criteria. (1) The lower bound on the expected FDR had to be less than or equal to 20% when the sensitivity was fixed at 80%, as calculated by using the formula derived in Box S1. (2) The p-value for the significance of the overlap between the bound and responsive gene sets had to be ≤ 0.01. For fixed threshold analysis, the p-value was calculated using the hypergeometric null distribution. For dual threshold analysis, it was calculated using the randomization-based null distribution, not the nominal p-value (see description of dual threshold optimization).

DUAL THRESHOLD OPTIMIZATION

Software availability

Software implementing dual threshold optimization and instructions can be found at https://github.com/BrentLab/Dual_Threshold_Optimization.

DTO algorithm

For each TF, dual threshold optimization (DTO) uses one binding location data set and one gene expression data set. The genes in each data set are ranked by the strength of their binding or expression signal. By default, the signal strength is the negative log p-value, but different experiments and methods may require different calculations of signal strength (see sections below). For each data set, DTO chooses a threshold on the ranks such that genes ranking above the threshold are considered positives for binding or response (see Fig. 2C). A series of rank-threshold combinations (places where the gray lines cross in Fig. 2C) are used to generate positive subsets of genes in each dataset. The series of thresholds for each data set, $T_1, T_2, \ldots$, were generated using the recurrence:

$$T_1 = 1$$
$$T_n = Floor(T_{n-1} * 1.01 + 1)$$

This formula produces a fine spacing among smaller subsets that becomes coarser as the subsets grow. If a threshold would split a group of genes that all have the same score that threshold is skipped. For each pair of subsets, a hypergeometric p-value was computed using a hypergeometric survival function (Scipy's hypergeom.sf) with the following parameters:

k = # of genes in the intersection of the subsets - 1
M = # of genes in the universe of assayed genes
n = # of genes in the expression subset
N = # of genes in the bound subset

This hypergeometric p-value is the probability of an intersection as large as, or larger than, the observed intersection, when choosing random subsets of genes, with the number of genes in each random subset equal to the number of genes in the positives defined by the rank threshold pair. We refer to this as the *nominal p-value* because it is only used for selecting the best pair of thresholds, not for determining whether the resulting overlap is significantly larger than would be expected by running DTO on random rankings. DTO returns the threshold combination that minimizes the nominal p-value.

Randomization-based p-values for overlaps identified by DTO

To produce a null distribution for testing the significance of the overlap chosen by DTO, a randomization procedure was used. A new set of data was generated using random assignment of signal strength scores to genes and then DTO was run. The best nominal p-value for each randomized data set was used to calculate a null distribution of nominal p-values. This was done by running the randomized DTO procedure 1000 times for each TF in each analysis and the distribution of nominal (hypergeometric) p-values enabled us to determine a P<0.01

significance threshold on the nominal p-value that is specific to that TF. When the nominal p-value of the rank pair chosen by DTO using the true data was below the threshold defined by the randomizations, the overlap was considered significant.

Application of DTO to yeast data

In the analysis of yeast data, the universe was defined as the set of all genes assayed in either of the two datasets being compared. For data sets that do not have p-values, the signal strength is the log fold change (ZEV) or score (NetProphet 2.0, GENIE3, Inferelator or MERLIN). For Calling cards, where many p-values were identical, ties were broken by the difference between the number of insertions in the experimental sample and the number of insertions in the control sample. The number of insertions was normalized to the total insertion count in each sample.

Occasionally, DTO can produce implausible results, such as concluding that all genes are responsive to a perturbation. To prevent this, we set very relaxed limits on the bound or responsive genes in certain data sets. For Harbison ChIP, we required $P<0.1$. For each inferred network output we required that the score of the TF-target relationship be among the top 150,000 scores. These were sufficient to eliminate any anomalous results; no constraints on the TFKO or ZEV data were required.

Application of DTO to human ENCODE data

In the analysis of human data on K562 cells, the universe was defined as the set of all genes detected in the gene expression dataset. Response signal strength for DTO was the absolute value of the log fold change, relative to non-perturbed control samples. DTO was limited to choosing bound or responsive genes with $P<=0.1$. For each inferred network output, the score of the TF-target relationship was required to be among the top 500,000 scores.

Application of DTO to human HEK293 data

In the analysis of human data on HEK293 cells, the universe was defined as the set of all genes detected in the gene expression dataset. Response signal strength for DTO was the absolute value of the log fold change, relative to non-perturbed control samples. No replicates or p-values were available for most TFs. For both raw perturbation-response data and inferred network scores, the score of the TF-target relationship was required to be among the top 300,000 scores.

COMPARISONS AMONG BINDING DATA SETS

Harbison ChIP-chip compared to Venters ChIP-chip

The TFs used in the comparison shown in Figure 4A are: Ash1, Cha4, Cin5, Fkh1, Fkh2, Gal4, Gcn4, Gln3, Ino4, Leu3, Msn2, Pho2, Rfx1, Rph1, Sfp1, Skn7, Stp1, Swi5, Uga3, Wtm1, Wtm2, Xbp1, Yap5, Yap6, Zap1, Zms1

Harbison ChIP-chip compared to ChIP-exo

The TFs used in the comparison shown in Figure 4B are:

Cat8, Cbf1, Ert1, Gal4, Gcn4, Gcr2, Hap1, Ino4, Leu3, Oaf1, Phd1, Pip2, Rds2, Rgt1, Rtg1, Rtg3, Sip4, Stb5, Sut1, Tye7

Harbison ChIP-chip compared to transposon calling cards
The TFs used in the comparison shown in Figure 4C are: Cbf1, Cst6, Gal4, Gcn4, Gcr2, Ino4, Leu3, Rgm1, Rgt1, Sfp1, Tye7, Zap1

RANK RESPONSE PLOTS
To create the lines in the rank response plots such Fig. 5A, we first determined the minimum of the number of responsive genes in the TFKO and the ZEV15 data -- call it *n*. A gene was considered responsive in the TFKO data if it had adjusted $P<0.05$ and in the ZEV15 data if it had shrunken absolute log fold change > 0. We then labeled the top *n* most strongly responsive genes in the TFKO and ZEV15 data as responsive for purposes of this plot (see "DTO algorithm" above for definitions of signal strength). This equalized the number of ZEV15-responsive and TFKO-responsive genes for each TF. We then sorted genes by the strength of their binding signal for the TF in question. Next, we considered the top 1, 2, 3, 4, etc. most strongly bound genes. For each such group, we calculated and plotted the fraction of genes that were responsive. For the mean rank-response plot (Fig. 5B) we simply averaged the response rates across the 12 TFs. In comparison of ChIP-chip, ChIP-exo and calling cards (Fig. 5C), we averaged the response rates across the 8 TFs that are present in all 3 binding data sets.

GO ENRICHMENT ANALYSIS
Gene ontology (GO) enrichments for each TF were analyzed using two networks mapped using different methods: (1) fixed threshold on Harbison ChIP data and TFKO data; (2) DTO on calling cards data and output from NetProphet 2.0 run on the TFKO and ZEV response data. For each TF, its target set in network 2 was the union of the output of DTO applied to NetProphet scores from ZEV expression data and DTO applied to NetProphet scores for TFKO expression data. The mapping of GO term to gene for *Saccharomyces cerevisiae* was queried using R Bioconductor library org.Sc.sgd.db (V3.5.0). Any GO terms annotated with less than 3 or greater than 300 genes were eliminated. Using the target genes of a TF identified from network (1) or (2), the GO enrichment in biological process was analyzed using the hypergeometric test implemented in R Bioconductor library GOstats (V2.44.0). The output p-values were used for ranking the enriched terms, from most significant to the least significant. When plotting the top GO term shown in Figure 4D, for each TF, we combined all terms from both networks into a single rank list. If multiple terms were enriched by the same set of targets, only the most specific term was retained, based on the GO hierarchical structure, i.e. redundant ancestral terms were removed from the rank list. Subsequently, the top GO term was chosen for the corresponding TF. When plotting the top GO terms shown in Figure S6, we used the GO terms enriched in one network and chose the top five (if available) as described above.

REFERENCES

Bergenholm, D, Liu, G, Holland, P, Nielsen, J. 2018. Reconstruction of a Global Transcriptional Regulatory Network for Control of Lipid Metabolism in Yeast by Using Chromatin

Immunoprecipitation with Lambda Exonuclease Digestion. *mSystems,* **3**. doi: 10.1128/mSystems.00215-17
Engel, SR, Dietrich, FS, Fisk, DG, Binkley, G, Balakrishnan, R, Costanzo, MC, Dwight, SS, Hitz, BC, Karra, K, Nash, RS, et al. 2014. The reference genome sequence of Saccharomyces cerevisiae: then and now. *G3 (Bethesda),* **4**: 389-398. doi: 10.1534/g3.113.008995
Fishilevich, S, Nudel, R, Rappaport, N, Hadar, R, Plaschkes, I, Iny Stein, T, Rosen, N, Kohn, A, Twik, M, Safran, M, et al. 2017. GeneHancer: genome-wide integration of enhancers and target genes in GeneCards. *Database (Oxford),* **2017**. doi: 10.1093/database/bax028
Greenfield, A, Hafemeister, C, Bonneau, R. 2013. Robust data-driven incorporation of prior knowledge into the inference of dynamic regulatory networks. *Bioinformatics,* **29**: 1060-1067. doi: 10.1093/bioinformatics/btt099
Hackett, SR, Baltz, EA, Coram, M, Wranik, BJ, Kim, G, Baker, A, Fan, M, Berndl, M, McIsaac, RS. 2019. Time-resolved genome-scale profiling reveals a causal expression network. *BioRxiv*. doi: https://doi.org/10.1101/619577
Harbison, CT, Gordon, DB, Lee, TI, Rinaldi, NJ, Macisaac, KD, Danford, TW, Hannett, NM, Tagne, JB, Reynolds, DB, Yoo, J, et al. 2004. Transcriptional regulatory code of a eukaryotic genome. *Nature,* **431**: 99-104. doi: 10.1038/nature02800
Holland, P, Bergenholm, D, Borlin, CS, Liu, G, Nielsen, J. 2019. Predictive models of eukaryotic transcriptional regulation reveals changes in transcription factor roles and promoter usage between metabolic conditions. *Nucleic Acids Res*. doi: 10.1093/nar/gkz253
Huynh-Thu, VA, Irrthum, A, Wehenkel, L, Geurts, P. 2010. Inferring regulatory networks from expression data using tree-based methods. *PLoS One,* **5**. doi: 10.1371/journal.pone.0012776
Imbeault, M, Helleboid, PY, Trono, D. 2017. KRAB zinc-finger proteins contribute to the evolution of gene regulatory networks. *Nature,* **543**: 550-554. doi: 10.1038/nature21683
Kang, Y, Liow, HH, Maier, EJ, Brent, MR. 2017. NetProphet 2.0: Mapping Transcription Factor Networks by Exploiting Scalable Data Resources. *Bioinformatics,* **34**: 249-257. doi: 10.1093/bioinformatics/btx563
Kemmeren, P, Sameith, K, van de Pasch, LA, Benschop, JJ, Lenstra, TL, Margaritis, T, O'Duibhir, E, Apweiler, E, van Wageningen, S, Ko, CW, et al. 2014. Large-scale genetic perturbations reveal regulatory networks and an abundance of gene-specific repressors. *Cell,* **157**: 740-752. doi: 10.1016/j.cell.2014.02.054
Rhee, HS, Pugh, BF. 2011. Comprehensive genome-wide protein-DNA interactions detected at single-nucleotide resolution. *Cell,* **147**: 1408-1419. doi: 10.1016/j.cell.2011.11.013
Rossi, MJ, Lai, WKM, Pugh, BF. 2018a. Genome-wide determinants of sequence-specific DNA binding of general regulatory factors. *Genome Res,* **28**: 497-508. doi: 10.1101/gr.229518.117
Rossi, MJ, Lai, WKM, Pugh, BF. 2018b. Simplified ChIP-exo assays. *Nat Commun,* **9**: 2842. doi: 10.1038/s41467-018-05265-7
Roy, S, Lagree, S, Hou, Z, Thomson, JA, Stewart, R, Gasch, AP. 2013. Integrated module and gene-specific regulatory inference implicates upstream signaling networks. *PLoS Comput Biol,* **9**: e1003252. doi: 10.1371/journal.pcbi.1003252
Schmitges, FW, Radovani, E, Najafabadi, HS, Barazandeh, M, Campitelli, LF, Yin, Y, Jolma, A, Zhong, G, Guo, H, Kanagalingam, T, et al. 2016. Multiparameter functional diversity of human C2H2 zinc finger proteins. *Genome Res,* **26**: 1742-1752. doi: 10.1101/gr.209643.116
Shively, CA, Liu, J, Chen, X, Loell, K, Mitra, RD. 2019. Homotypic cooperativity and collective binding are determinants of bHLH specificity and function. *Proc Natl Acad Sci U S A,* **116**: 16143-16152. doi: 10.1073/pnas.1818015116

Venters, BJ, Wachi, S, Mavrich, TN, Andersen, BE, Jena, P, Sinnamon, AJ, Jain, P, Rolleri, NS, Jiang, C, Hemeryck-Walsh, C, et al. 2011. A comprehensive genomic binding map of gene and chromatin regulatory proteins in *Saccharomyces*. *Mol Cell,* **41**: 480-492. doi: 10.1016/j.molcel.2011.01.015

Wang, H, Mayhew, D, Chen, X, Johnston, M, Mitra, RD. 2011. Calling Cards enable multiplexed identification of the genomic targets of DNA-binding proteins. *Genome Res,* **21**: 748-755. doi: 10.1101/gr.114850.110

Wang, H, Mayhew, D, Chen, X, Johnston, M, Mitra, RD. 2012. "Calling cards" for DNA-binding proteins in mammalian cells. *Genetics,* **190**: 941-949. doi: 10.1534/genetics.111.137315

Weirauch, MT, Yang, A, Albu, M, Cote, AG, Montenegro-Montero, A, Drewe, P, Najafabadi, HS, Lambert, SA, Mann, I, Cook, K, et al. 2014. Determination and inference of eukaryotic transcription factor sequence specificity. *Cell,* **158**: 1431-1443. doi: 10.1016/j.cell.2014.08.009
